# Inhibition of NEK7 Suppressed Hepatocellular Carcinoma Progression by Mediating Cancer Cell Pyroptosis

**DOI:** 10.3389/fonc.2022.812655

**Published:** 2022-02-10

**Authors:** Zilong Yan, Qingen Da, Zhangfu Li, Qirui Lin, Jing Yi, Yanze Su, Guanyin Yu, Qingqi Ren, Xu Liu, Zewei Lin, Jianhua Qu, Weihua Yin, Jikui Liu

**Affiliations:** ^1^ Department of Hepatobiliary Surgery, Peking University Shenzhen Hospital, Shenzhen-Peking University-Hong Kong University of Science and Technology Medical Center, Shenzhen, China; ^2^ Department of Hepatobiliary Surgery, Peking University Shenzhen Hospital, Shenzhen, China; ^3^ Department of Cardiovascular Surgery, Peking University Shenzhen Hospital, Shenzhen-Peking University-Hong Kong University of Science and Technology Medical Center, Shenzhen, China; ^4^ Department of Pathology, Peking University Shenzhen Hospital, Shenzhen, China

**Keywords:** NEK7, hepatocellular carcinoma, pytoptosis, cancer–stromal interaction, migration, invasion

## Abstract

NIMA-related kinase 7 (NEK7) is a serine/threonine kinase involved in cell cycle progression *via* mitotic spindle formation and cytokinesis. It has been related to multiple cancers, including breast cancer, hepatocellular cancer, lung cancer, and colorectal cancer. Moreover, NEK7 regulated the NLRP3 inflammasome to activate Caspase-1, resulting in cell pyroptosis. In the present study, we investigated whether NEK7 is involved in cell pyroptosis of hepatocellular carcinoma (HCC). Interestingly, we found that NEK7 was significantly related to expression of pyroptosis marker GSDMD in HCC. We found that NEK7 expression was significantly correlated with GSDMD expression in bioinformatics analysis, and NEK7 expression was significantly co-expressed with GSDMD in our HCC specimens. Cell viability, migration, and invasion capacity of HCC cell lines were inhibited, and the tumor growth in the xenograft mouse model was also suppressed following knockdown of NEK7 expression. Mechanistic studies revealed that knockdown of NEK7 in HCC cells significantly upregulated the expression of pyroptosis markers such as NLRP3, Caspase-1, and GSDMD. Coculture of HCC cells stimulated hepatic stellate cell activation by increasing p-ERK1/2 and α-SMA. Knockdown of NEK7 impaired the stimulation of HCC cells. Therefore, downregulation of NEK7 inhibited cancer–stromal interaction by triggering cancer cell pyroptosis. Taken together, this study highlights the functional role of NEK7-regulated pyroptosis in tumor progression and cancer–stromal interaction of HCC, suggesting NEK7 as a potential target for a new therapeutic strategy of HCC treatment.

## Introduction

Hepatocellular carcinoma (HCC) is the most common primary liver cancer and the second most common cause of cancer deaths worldwide (1), with a 5-year overall survival rate of 12% and a median survival of 23 months for Chinese HCC patients (2). Early-stage HCC may be treated with surgical resection and systemic therapy. However, treatment is associated with high overall relapse rates, and curative resection is not feasible after tumor progressed (3). More unfortunately, >70% of patients with HCC are diagnosed at the late stage (4, 5). As curative resection and chemotherapy are not feasible, it is urgent to explore non-surgical therapeutic approaches for new biomarkers and effective therapeutic strategies (6).

NIMA-related kinases (NEKs) of NEK1 to NEK11 are serine/threonine kinases that drive cell-cycle dynamics by modulating mitotic spindle formation and cytokinesis (7). Among the NEK family members, the smallest NEK7 and NEK6 share more than 85% sequence identity (8). Previous studies reported overexpression of NEK7 in human cancer, including head and neck (9), breast (10), pancreas (11), liver (12), and colorectum and lung (13). Moreover, the biological role of NEK7 was not limited to cell proliferation capacity; involvement of NEK7 in tumorigenesis and metastasis was also proved (9–12).

Recent studies have shown that ROS, potassium efflux, and other factors activate Caspase-1 by regulating the interaction of NEK7 and NLRP3 inflammasome, leading to cell pyroptosis (14–16). Pyroptosis is an inflammatory form of programmed cell death, mainly mediated by the inflammasome to activate a variety of cysteine proteases including Caspase-1/4/5/11, resulting in pyroptosis execution of protein Gasdermin D (GSDMD) cleavage. The pore-forming activity of GSDMD leads to cell perforation, changing the osmotic pressure of the cell membrane and losing its integrity, and then mediating the maturation and secretion of IL-1β and IL-18 (17, 18). Accumulating studies of NEK7 or pyroptosis were reported individually in breast cancer, colorectal cancer, lung cancer, and liver cancer (12, 13, 19–21). However, the relationship between NEK7 and pyroptosis in HCC and the biological role and underlying mechanisms of pyroptosis in HCC remain unclear.

In the present study, by using bioinformatic databases, cell lines, and tumor samples, we investigated the correlation between NEK7 expression and GSDMD expression in human HCC. We detected a significant correlation of NEK7 in the migration and invasion ability of HCC cell lines. Moreover, NEK7 inhibited hepatic stellate cell (HSC) activation and cancer–stromal interaction through regulation of cancer cell pyroptosis. In a splenic xenograft mouse model, knockdown of NEK7 expression suppressed HCC primary tumor formation. Therefore, the potential of NEK7-targeted treatment for cancer–stromal interaction of HCC may be possible.

## Materials and Methods

### HCC Tissues and Ethical Approval

The HCC specimens were obtained from 12 patients who underwent hepatectomy for liver cancer in Peking University Shenzhen Hospital from 2011 to 2014. For immunohistochemistry staining, tissues were embedded, sliced, and stained at the Pathology Department of Peking University Shenzhen Hospital. The tissues were snap frozen in liquid nitrogen immediately after resection and stored at −80°C until RNA extraction. Paired HCC tumor and tumor-adjacent normal tissues were used to examine mRNA expression levels in our study. Experiments involving human samples have been approved by the committee for ethical review of research involving human subjects at Peking University and Peking University Shenzhen Hospital. All patients signed informed consent for participation in the study. The clinicopathological characteristics of all patients in this study are summarized in **Table 1**.

**Table 1:** The primer sequences used for quantitative real-time PCR.

Patient number	Sex	Age	Admission date	Admission date	Date of surgery	Diagnosis	Surgical procedure	Other diagnosis	Pathological diagnosis
164501	Male	Y39	1/18/2011 0:00	1/29/2011 0:00	1/21/2011 0:00	Hepatocellular carcinoma	Open resection of liver lesions	Chronic hepatitis B	Moderately differentiated hepatocellular carcinoma
219402	Male	Y37	3/20/2011 0:00	4/6/2011 0:00	3/25/2011 0:00	Hepatocellular carcinoma	Open resection of liver lesions	Post-hepatitic cirrhosis, chronic hepatitis B	Moderately differentiated hepatocellular carcinoma
51921	Male	Y64	8/5/2011 0:00	8/23/2011 0:00	8/9/2011 0:00	Hepatocellular carcinoma	Open resection of liver lesions	Hepatic cyst, renal cyst (acquired)	Atypical hyperplasia of liver tissue in the right lobe of the liver
261879	Female	Y36	10/4/2012 0:00	10/22/2012 0:00	10/13/2012 0:00	Hepatocellular carcinoma	Open resection of liver lesions	Chronic cholecystitis, gallbladder polyps	Hepatocellular carcinoma; chronic cholecystitis with cholesterol polyps
32118	Male	Y67	10/20/2012 0:00	11/11/2012 0:00	10/24/2012 0:00	Hepatocellular carcinoma	Open resection of liver lesions	Decompensated liver cirrhosis, hypoproteinemia, malignant hypertension, chronic hepatitis C, hydronephrosis, nephrarctia, hepatic cyst, moderate anemia	Moderately differentiated hepatocellular carcinoma
311931	Male	Y60	6/3/2014 11:14	6/16/2014 0:00	6/10/2014 0:00	Hepatocellular carcinoma	Laparoscopic hepatic lobectomy	Chronic hepatitis B, chronic hepatitis B	Moderately differentiated hepatocellular carcinoma of the left outer lobe of liver; chronic cholecystitis hepatocellular carcinoma
28516	Male	Y55	7/14/2014 15:06	8/4/2014 10:00	7/24/2014 0:00	Hepatocellular carcinoma	Open resection of liver lesions	Chronic hepatitis B	Moderately differentiated hepatocellular carcinoma of the right outer lobe of liver
317143	Male	Y48	7/31/2014 16:15	8/18/2014 17:38	8/8/2014 0:00	Hepatocellular carcinoma	Laparoscopic liver lesion resection	Cholecystolithiasis, chronic cholecystitis, chronic hepatitis B	moderately differentiated hepatocellular carcinoma of the right posterior lobe; chronic cholecystitis, cholecystolithiasis
324741	Male	Y59	10/29/2014 14:32	11/13/2014 10:00	11/3/2014 0:00	Hepatocellular carcinoma	Laparoscopic liver lesion resection	Kidney stone, hypertension, diabetes, benign prostatic hyperplasia	Moderately differentiated hepatocellular carcinoma of the right posterior lobe; chronic cholecystitis
326792	Female	Y67	11/21/2014 15:05	12/2/2014 16:15	11/27/2014 0:00	Hepatocellular carcinoma	Laparoscopic hepatic lobectomy	HbsAg carrier, chronic cholecystitis	Chronic cholecystitis; moderately differentiated hepatocellular carcinoma of left lateral lobe of liver
327691	Male	Y59	12/2/2014 8:40	12/10/2014 10:00	12/4/2014 0:00	Hepatocellular carcinoma	Laparoscopic liver lesion resection	Liver cirrhosis, cholecystolithiasis, HbsAg carrier	Hepatocellular carcinoma
327830	Male	Y59	12/3/2014 14:21	12/31/2014 9:00	12/12/2014 8:00	Hepatocellular carcinoma	Open resection of liver lesions	bile leakage, lumbar disc herniation	Moderately differentiated hepatocellular carcinoma

### Immunohistochemistry

Immunohistochemistry (IHC) staining was performed in tissue sections as described previously (22). Sections were incubated with goat anti-NEK7 (ab166776, Abcam, Cambridge, MA, USA) and anti-GSDMD (ab219800, Abcam) overnight at 4°C and stained with a second antibody by using an SP-POD Kit according to the manufacturer’s instructions (#SP0041, Solarbio, Beijing, China). Multiplexed IHC (mIHC) was performed using a multiple fluorescent immunohistochemical staining kit (abs50012, Absin, Shanghai, China) according to the manufacturer’s instructions (23). Sections were observed using an optical microscope (BX53, Olympus, Tokyo, Japan).

### Cell Lines, Culture Conditions, and Treatment

The following HCC cell lines were used in this study: Hep3B, Huh7, HepG2, MHCC97L, MHCC97H, and L02 (Chinese Academy of Medical Sciences, Beijing, China). All cell lines were maintained in Dulbecco’s modified Eagle’s medium (DMEM; Gibco, Grand Island, NY, USA) and supplemented with 10% fetal bovine serum (Gibco, Grand Island, NY, USA), 100 U/ml penicillin, and 100 U/ml streptomycin (Invitrogen, Carlsbad, CA, USA) at 37°C in a humidified atmosphere with 5% CO_2_. For inducing pyroptosis, cells (2 × 10^5^ cells/well in 6-well culture plates) were treated with 1 μg/ml lipopolysaccharide (L2630, Sigma-Aldrich) for 4 h and then cells were treated with 10 μM nigericin (HY-100381, MedChemExpress, Princeton, NJ, USA) for 2 h before subsequent analyses (24).

### Enzyme-Linked Immunosorbent Assay (ELISA) of IL-1β

The medium of each treatment group was collected and centrifuged at 1,000 g for 20 min at 4°C to remove impurities and cell debris, according to the instructions of Human IL-1 Beta ELISA Kit (ELH-IL1b, RayBio, Peachtree Corners, GA, USA). The absorbance of the microplate reader was set to 450 nm, and the standard curve was generated using a standard product to determine the concentration of each culture of IL-1 in the supernatant. Three separate preparations were used, and each experiment was carried out in triplicate wells and repeated 3 times.

### Lactate Dehydrogenase Release Assay

The lactate dehydrogenase (LDH) assay was performed according to the LDH Cytotoxicity Assay Kit (C0016, Beyotime, Shanghai, China). The cells were seeded on 96-well plates for the logarithmic growth phase and divided into a negative control group, maximum active well, and experiment-indicated groups. The LDH release reagent was added 1 h before the scheduled time. The 96-well plate was centrifuged at 400 g for 5 min. Then, 120 μl of the supernatant was aspirated into a new 96-well plate, before measuring the absorbance at a wavelength of 490 nm using a microplate reader to calculate the LDH content. Each experiment was performed in triplicate and repeated three times.

### Quantitative RT-PCR

Total RNA was extracted from cultured cells using a High Pure RNA Isolation Kit (Roche Diagnostics, Mannheim, Germany) and DNase I (Roche Diagnostics, Sigma-Aldrich) treatment according to the manufacturer’s instructions. RNA was directly reverse transcribed using the HiScript^®^ II 1st Strand cDNA Synthesis Kit (MR101-01, Vazyme, Nanjing, China) according to the manufacturer’s instructions. Quantitative RT-PCR (qRT-PCR) was performed using the AceTaq^®^ qPCR SYBR Green Master Mix (Q121-03, Vazyme, China). We designed several specific primer sequences (Gene Pharma, China). For PCR analysis, three independent experimental repeats were performed, and data are presented as mean ± standard error of mean. Primer sequences are listed in **Table 2**. mRNA expression levels are presented as relative expression normalized to β-actin.

**Table 2:** The clinicopathological characteristics of HCC patients.

Sense (5′–3′)	Antisense (5′–3′)
1. NEK7 primer (human): CACCTGTTCCTCAGTTCCAAC	CTCCATCCAAGAGACAGGCTG
2. NEK7 primer (human): ACGTGCTGATTGCATCAAAG	GCACTGCAAAGCTGAACAAA
3. NEK7 primer (human): CCACTGGGGTGGTAAAACTTG	AAGGACTTTGTAATGCAGCCAT
4. NLRP3 primer (human): AGAAGAGACCACGGCAGAAG	CCTTGGACCAGGTTCAGTGT
5. GSDMD primer (human): CCAGTGCCTCCATGAATGTGT	TCACCACAAACAGGTCATCCC
6. β-Actin primer (human): AAGGAAGGCTGGAAGAGTGC	CTGGGACGACATGGAGAAAA

### Western Blot Analysis

Cells were prepared at 4°C in RIPA buffer (P0013J, Beyotime, China) containing proteinase inhibitor cocktail (B14001, Bimake, Houston, TX, USA) and phosphatase inhibitor cocktail (B15001, Bimake). Proteins were separated on 4%–20% precast mini polyacrylamide gels (SurePAGE™, GenScript, Nanjing, China) and transferred to PVDF membranes. The membranes were incubated overnight at 4°C with anti-NEK7 (ab95873, Abcam), anti-GSDMD (ab219800, Abcam), anti-cleaved N-terminal GSDMD ab215203, Abcam), anti-NLRP3 (ab263899, Abcam), anti-cleavage caspase-1 (4199S, Cell Signaling Technology, Danvers, MA, USA), anti-ERK1/2 (9102S, Cell Signaling Technology), anti-p-ERK1/2 (9101S, Cell Signaling Technology), anti-α-SMA (A17910, ABclonal, Woburn, MA, USA), and anti-β-actin (66009-1-Ig, Proteintech, Rosemont, IL, USA). Membranes were then probed with appropriate secondary antibodies (Cell Signaling Technology). Immunoblot signals were detected by a Millipore chemical developer (Millipore Sigma, Burlington, MA, USA).

### Matrigel Invasion and Migration Assays

The invasiveness and migration capacity of cancer cells were assessed by determining the number of cells invading or migrating across transwell chambers as previously described (25). Cancer cells were labeled with CellTracker Green CMFDA (40721ES50, Yeasen, Shanghai, China) according to the manufacturer’s instructions. For invasion assays, cells (1 × 10^5^ cells/250 μl) were seeded in the upper transwell chamber (8-μm pore size; Becton Dickinson, Franklin Lakes, NJ, USA) containing 100 ml of reconstituted matrigel-coated membrane (20 μg/well, BD Biosciences, Bedford, MA, USA) at 24 h after knockdown of NEK7. Cells were incubated for 48 h, and the number of invaded cancer cells was counted. Cell migration assays were performed using the same protocol as the invasion assay without a matrigel-coated membrane. Cells were allowed to migrate and counted 24 h after cell seeding into the upper chamber. In both assays and at each time point, invaded or migrated cells at the bottom of the chamber were determined in five random fields at ×100 magnification using a fluorescent microscope (Leica, DMi8, Wetzlar, Germany). Each experiment was performed in triplicate and repeated at least three times.

### Coculture Migration Assay

The migration capacity of coculture was assessed by wound healing assay. Cells were dyed with Cell Tracker Green or Red, then seeded in a culture insert (ibidi, 81176, Martinsried, Germany). After cell attachment, the culture insert was removed and cells were allowed to migrate. At each time point, the wound closure was measured (BX53, Olympus, Japan). Graphs show the quantification of closure length (reference to 0 h). For direct coculture of cancer cells and HSCs, cancer cells and HSCs were dyed with Cell Tracker Green or Red, mixed, and then seeded in the same culture insert. At the indicated time point, migrated cell number and distance were measured by using a fluorescent microscope (Leica, DMi8, Germany). Each experiment was performed in triplicate and repeated three times.

### Cell Viability Assay

Cells (1 × 10^3^ cells/well) were seeded in 96-well plates. Cell viability was examined using the CellTiter-Lumi™ Plus Cell Viability Assay Kit (C0068M, Beyotime, China) following the manufacturer’s instructions. Background was subtracted using values from wells containing only culture medium.

### Establishment of Small Hairpin RNA-Expressing Cells

Two NEK7 small-hairpin (shRNA, shNEK7-1: ccggatatgggctataataca, shNEK7-2: ctccgacagttagttaatatg) vectors (GenePharma, Shanghai, China) were transfected into MHCC97L cells according to the manufacturer’s instructions. Non-targeting shRNA (GenePharma) was used as control. Puromycin (S7417, Selleck Chemicals, Houston, TX, USA) was used to select NEK7-stably expressing clones. Selection was performed for more than 3 weeks. shRNA-mediated NEK7 knockdown was confirmed by quantitative RT-PCR and Western blot.

### *In Vivo* Experiments

BALB/c athymic female nude mice were purchased (GemPharmatech, Nanjing, China) and transported to our institution at 4 weeks of age. 10 mice were randomized divided into 2 groups. Mice were raised in a specific pathogen-free animal room (SPF) of Shenzhen Peking University, Hong Kong University of Science and Technology Medical Center. The Animal Experimentation Ethics Committee approved our study and ensured that all experiments conformed to all relevant regulatory standards. After 1 week of accustomization, 1 × 10^6^ MHCC97L cells were injected into the spleen of mice and liver metastasis was quantified after 6 weeks of cell implantation. Mice were cervically dislocated after anesthesia of 2% inhalable isoflurane, and the liver metastases were collected for the following evaluation.

### Statistical Analysis

The chi-squared test was performed to assess relationships between NEK7 protein expression and clinicopathological features. For results of *in vitro* experiments, values are expressed as the mean ± SEM. Comparisons of RT-QPCR were carried out using the Student’s t-test. Comparisons of RNA expression in bioinformatic analysis were performed using the Wilcox test and Kruskal–Wallis test. Kaplan–Meier analyses were compared using the log-rank test. *p* < 0.05 was used to define statistical significance. Statistical analysis was conducted using R language and GraphPad Prism.

## Results

### Expression of NEK7 Is Correlated to GSDMD in Bioinformatic Analysis of HCC

**Figure 1:**
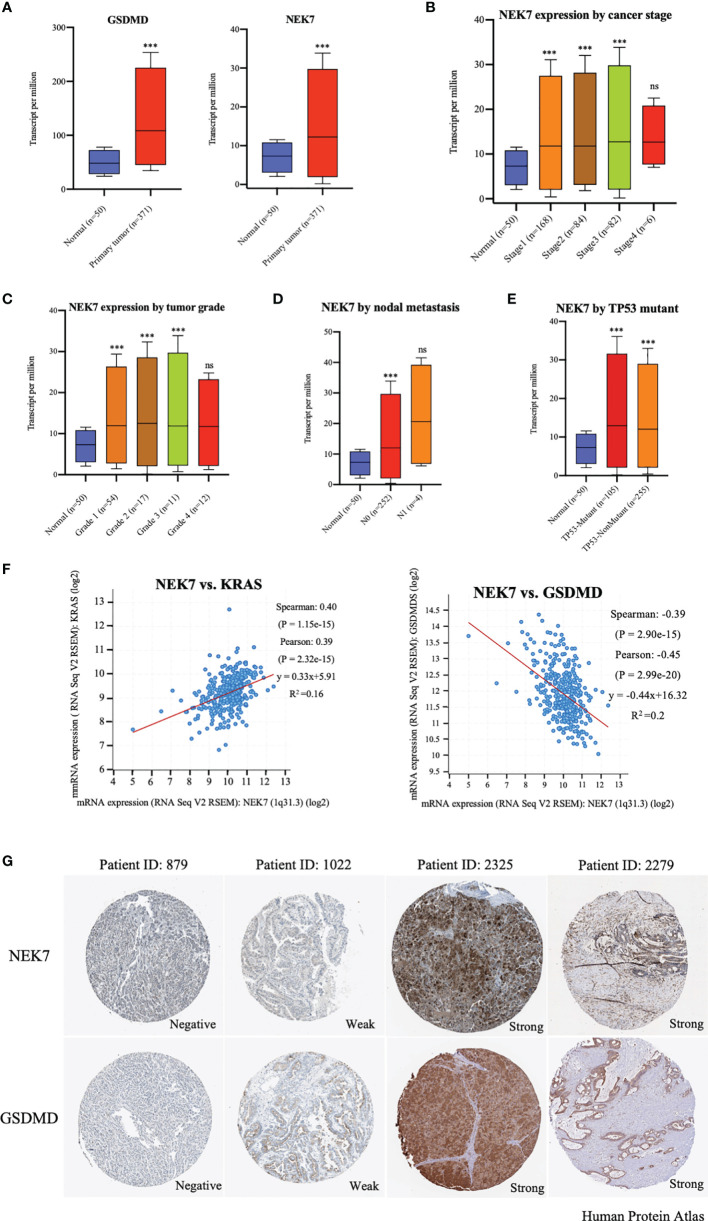
Expression of NEK7 correlated with GSDMD in hepatocellular carcinoma in bioinformatics analysis. **(A)** Expressions of NEK7 and GSDMD were significantly upregulated in tumor tissues compared to tumor-adjacent normal tissues. ***p < 0.001. **(B–E)** Expression of NEK7 significantly correlated with liver hepatocellular carcinoma cancer stage, tumor grade, nodal metastasis, and TP53 mutant. ***p < 0.001. ns, no significance. **(F)** Expression of NEK7 significantly correlated with Kras and GSDMD in HCC tissues. **(G)** Heterogenetic expressions of NEK7 and GSDMD in HCC tissues. NEK7 and GSDMD were co-expressed in the same tumor samples.

By analyzing UALCAN (http://ualcan.path.uab.edu/index.html) (26), we found that expressions of NEK7 and GSDMD were significantly upregulated in tumor tissues compared to tumor-adjacent normal tissues of liver hepatocellular carcinoma (**Figure 1A**). The expression of NEK7 in liver hepatocellular carcinoma was correlated with cancer stage, tumor grade, nodal metastasis, and TP53 mutant and histological subtypes (**Figures 1B–E** and **S1A**). Based on the bioinformatic analysis of the datasets from The Cancer Genome Atlas (TCGA, https://www.cancer.gov/tcga) database (27), we found that NEK7 expression was highly correlated with expression levels of Kras and GSDMD in HCC tumor tissues (**Figures 1F, G** and **S1B**). In primary tumors, heterogenetic expressions of NEK7 and GSDMD including negative, weak, and strong were observed using Human Protein Atlas (www.protenatlas.org) (28). Meanwhile, we found a co-expression relationship between NEK7 and GSDMD in human specimens (**Figure 1G**). Given the results of NEK7 and GSDMD in HCC, we speculated that the expression of NEK7 may regulate HCC cell pyroptosis and play a crucial role in cancer progression.

### NEK7 and GSDMD Is Overexpressed in HCC

**Figure 2:**
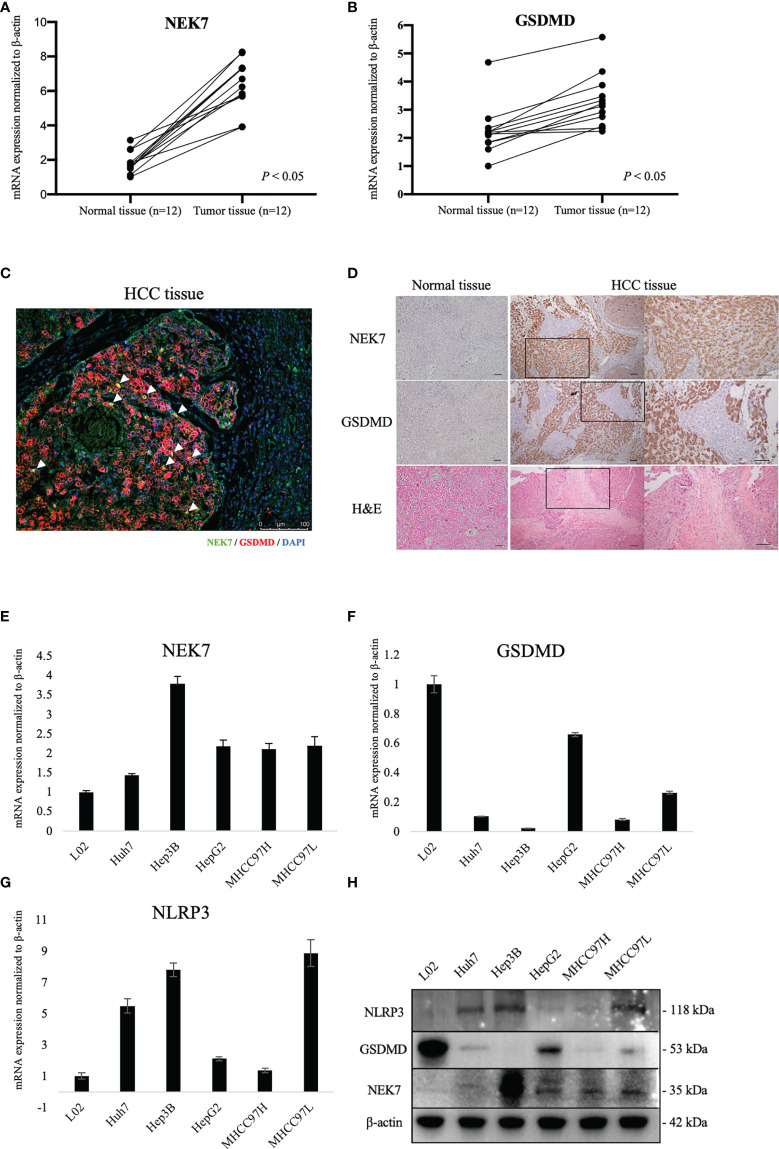
Expression of NEK7 and GSDMD in hepatocellular carcinoma tissues and cell lines. **(A, B)** mRNA expressions of NEK7 and GSDMD were significantly upregulated in tumor tissues compared to tumor-adjacent normal tissues. **(C)** mIHC staining of NEK7 (green) and GSDMD (red) on HCC specimens. Scale bar = 100 μm. **(D)** Co-locolization between NEK7 and GSDMD was observed on serial sections of HCC specimens. Scale bars = 100 μm. **(E)** NEK7 mRNA expression in HCC cell lines. **(F)** GSDMD mRNA expression in HCC cell lines. **(G)** NLRP3 mRNA expression in HCC cell lines. mRNA expression was normalized by β-actin expression. Error bar, error value in triplicate. **(H)** Protein expressions of NEK7, GSDMD, and NLRP3 in HCC cell lines.

By using fresh frozen samples of tumor and paired tumor-adjacent normal tissues from our institution, we found that NEK7 and GSDMD were significantly upregulated in tumor tissues compared to normal tissues (**Figures 2A, B** **)**. Then, we examined the expressions of NEK7 and GSDMD in normal liver tissue and HCC tissues. As negative expressions of NEK7 and GSDMD were found in normal liver tissue, we confirmed co-localization between NEK7 and GSDMD in multiple fluorescent immunohistochemical stainings (**Figures 2C** and **S2A**) and serial sections (**Figure 2D**) using HCC specimens from our institution. Moreover, we investigated mRNA (**Figures 2E–G**) and protein (**Figure 2H**) expressions of NEK7, as well as pyroptosis marker NLRP3 and GSDMD expression of HCC cell lines.

### The Biological Function of NEK7 in HCC *In Vitro*

**Figure 3:**
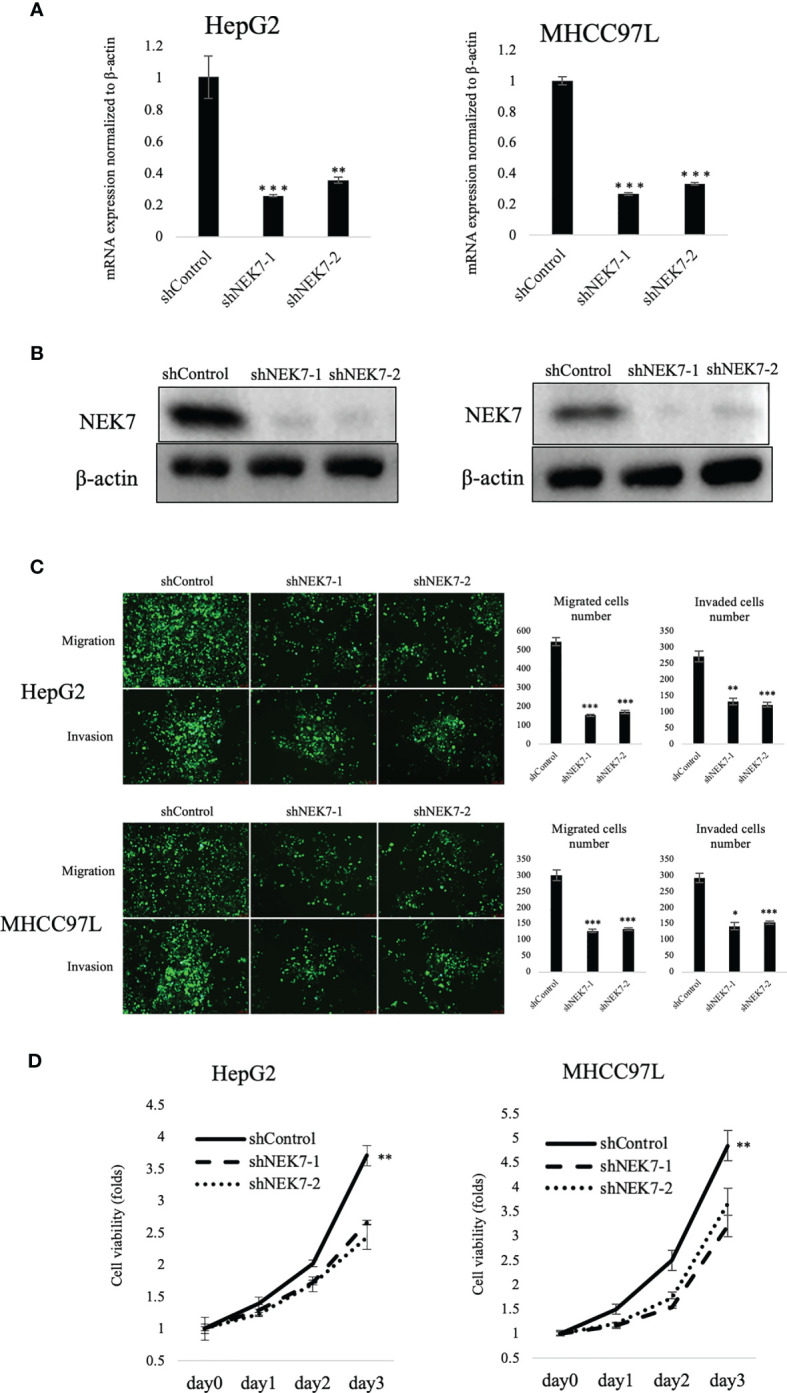
Knockdown of NEK7 reduces migration, invasion, and cell viability of HCCs. **(A, B)** qRT-PCR and Western blot of NEK7 mRNA and protein levels in cells transfected with shRNAs targeting NEK7 or negative control. ***p < 0.001. **(C)** shControl or shNEK7 cells were dyed with CellTracker Green; migration and invasion assays were performed for 24 and 48 h. Graphs show the quantification of cells calculated from five fields. Original magnification: ×100. Scale bars = 100 μm. *p < 0.05, **p < 0.01, ***p < 0.001. **(D)** Cell viability of cells was determined by CellTiter-Lumi™ Plus cell viability assay. **p < 0.01.

To explore the biological role of NEK7 in HCCs, we investigated the effect of NEK7 knockdown on the invasiveness and migration activities of MHCC97L and HepG2 cells due to their overexpression of NEK7 and GSDMD. Downregulation of NEK7 expression in HepG2 and MHCC97L cells was achieved stably and efficiently by using short hairpin RNA transfection (**Figures 3A, B** **)**. Cells were labeled with green fluorescence, and then transwell migration and invasion assay were performed. In result, the migration and invasion abilities of cancer cells were significantly decreased after NEK7 knockdown compared to the control group (**Figure 3C**). Meanwhile, cell proliferation was significantly decreased following downregulation of NEK7 expression (**Figure 3D**).

### NEK7 Regulated Cancer Cell Pyroptosis and Inhibited Its Migratory Toward HSC

We next investigated the effect of NEK7 in the cancer–stromal interaction of HCC (29, 30).

**Figure 4:**
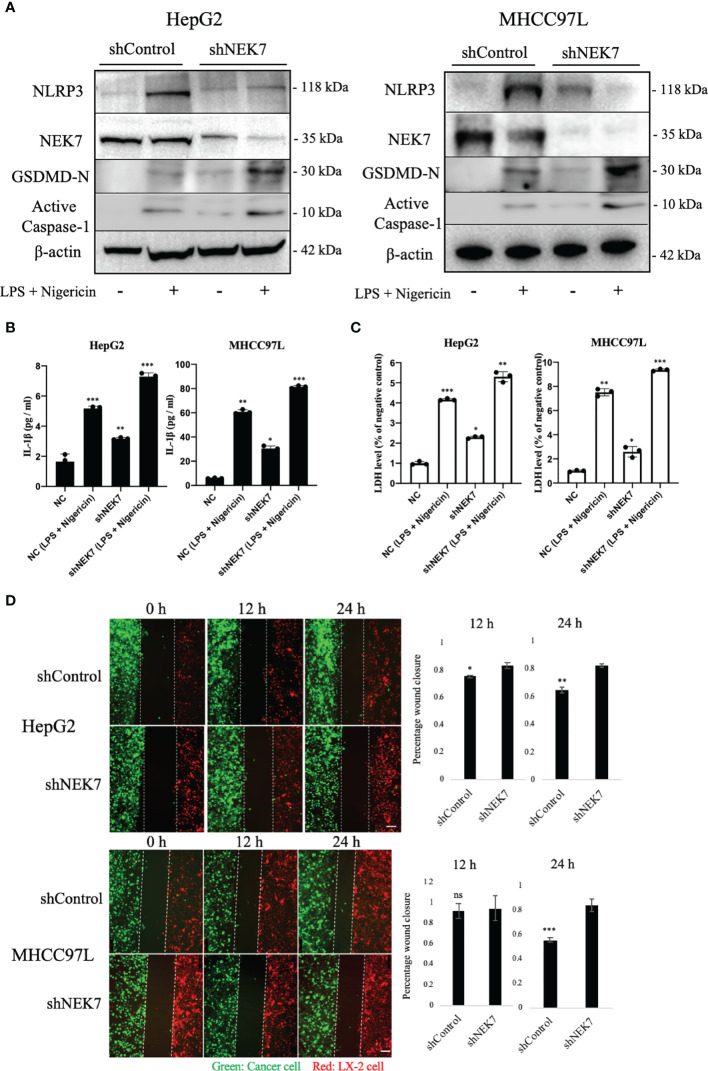
NEK7 regulated HCC pyroptosis and inhibited its migratory toward HSC. **(A)** Protein levels of NLRP3, GSDMD, NEK7, and Cleaved caspase-1 in HCCs transfected with shRNAs targeting NEK7 or negative control. LPS and nigericin treatment was positive control for cell pyroptosis. **(B)** IL-1β release of HCC supernatant. **(C)** LDH release of HCC supernatant. **(D)** shControl or shNEK7 cells were dyed with CellTracker Green, LX-2 cells were dyed with CellTracker Red, and migration was performed for 12 and 24 h. Original magnification: ×100. Scale bars = 100 μm. *p < 0.05, **p < 0.01, ***p < 0.001.

Lipopolysaccharide (LPS) combined with nigericin was reported to induce pyroptosis (24, 31); our results showed that treatment of LPS and nigericin could induce HCC cell pyroptosis by remarkably upregulating the expression of active Caspase-1 and N-terminal GSDMD which are commonly used markers of pyroptosis. Compared to control, independent knockdown of NEK7 expression slightly upregulated the expressions of pyroptosis-related genes NLRP3, active Caspase-1, and N-terminal GSDMD (**Figure 4A**). The occurrence of pyroptosis was confirmed by investigating the IL-1β and LDH release (**Figures 4B, C** **)**. Then we conducted a wound healing assay to investigate the cancer–stromal interaction by using cancer cells and human hepatic stellate cell line LX-2 cells. We found that knockdown of NEK7 in cancer cells inhibited the cancer–stromal interaction by repression of cell migratory toward LX-2 cells (**Figure 4D**).

### NEK7-Regulated HCC Cell Pyroptosis Inhibited HSC Activation and Cancer–Stromal Interaction

**Figure 5:**
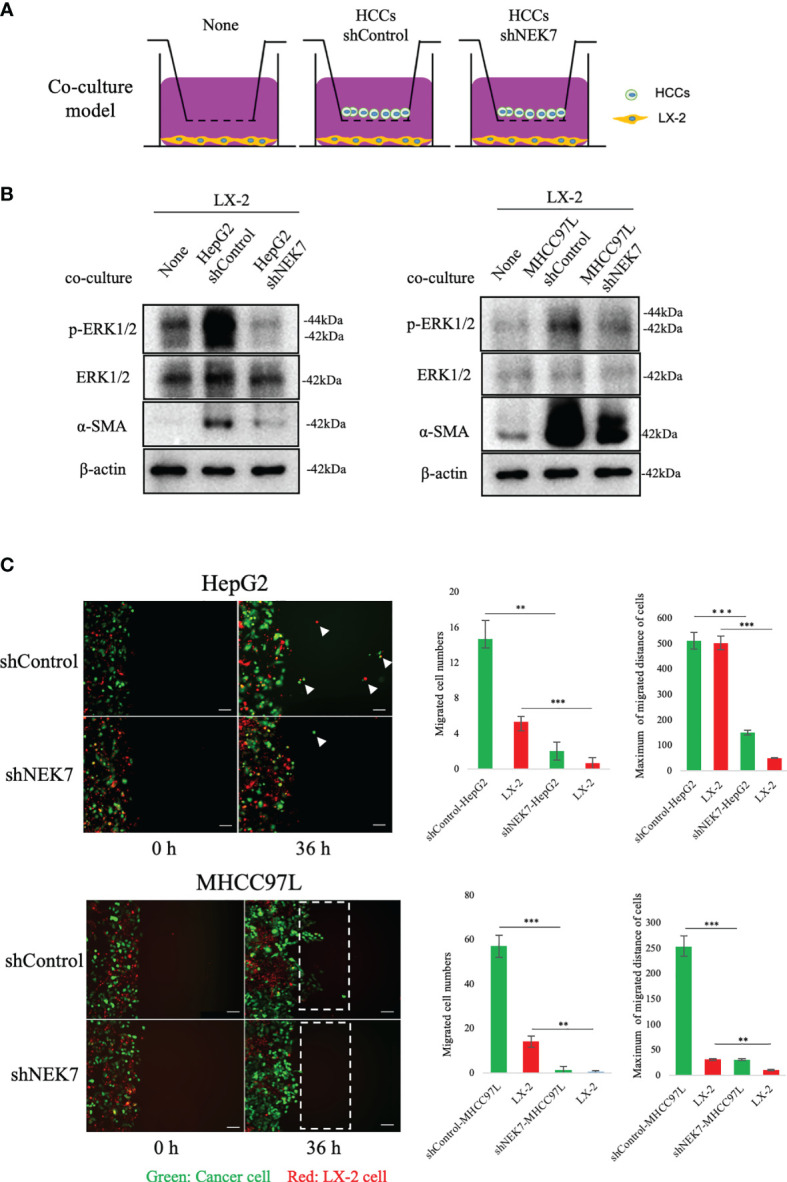
NEK7 promoted cancer–stromal interaction by activating HSC. **(A)** In indirect coculture experiments, first HSCs were seeded, and 24 h later, medium was replaced and transwell chambers (3-μm pores; Becton Dickinson) were placed in 24-well dishes, and then cells were seeded into the transwell chambers. After incubation for 2 days, HSCs in the lower chamber were collected and total protein was extracted. **(B)** Western blot of ERK1/2, p-ERK1/2, and α-SMA levels in HSCs, alone, or after coculture with HCCs. **(C)** shControl or shNEK cells direct the coculture model, coculture promoted the migration of both cancer cells and HSCs, meanwhile downregulation of NEK7 impaired the migration ability. Original magnification: ×100. Scale bars = 100 μm. **p < 0.01, ***p < 0.001.

Cancer–stromal interaction plays an important role in HCC progression (32). Cancer cells activated HSC and promoted tumor-stromal interaction (33, 34). By using the transwell indirect coculture system (**Figure 5A**), we confirmed that coculture of cancer cells significantly stimulated LX-2 activation by upregulating the expressions of α-SMA and p-ERK1/2 (**Figure 5B**). α-SMA and p-ERK1/2 were reported as markers of HSC activation (32, 35); coculture of shNEK7-cells impaired the activation of α-SMA or p-ERK1/2 in LX-2 cells compared to the positive control (**Figure 5B**). Next, we mixed cancer cells and HSCs directly and monitored the migration of both cancer cells and HSCs. Knockdown of NEK7 significantly reduced migrated cell numbers and maximum migrated distance of both cancer cells and HSCs (**Figure 5C**). As knockdown of NEK7 alone was sufficient to induce pyroptosis by mediating GSDMD-N and IL-1β release, these results suggested that NEK7-regulated HCC cell pyroptosis inhibited HSC activation and cancer–stromal interaction.

### Downregulation of NEK7 Suppressed HCC Primary Tumor Formation *In Vivo*

**Figure 6:**
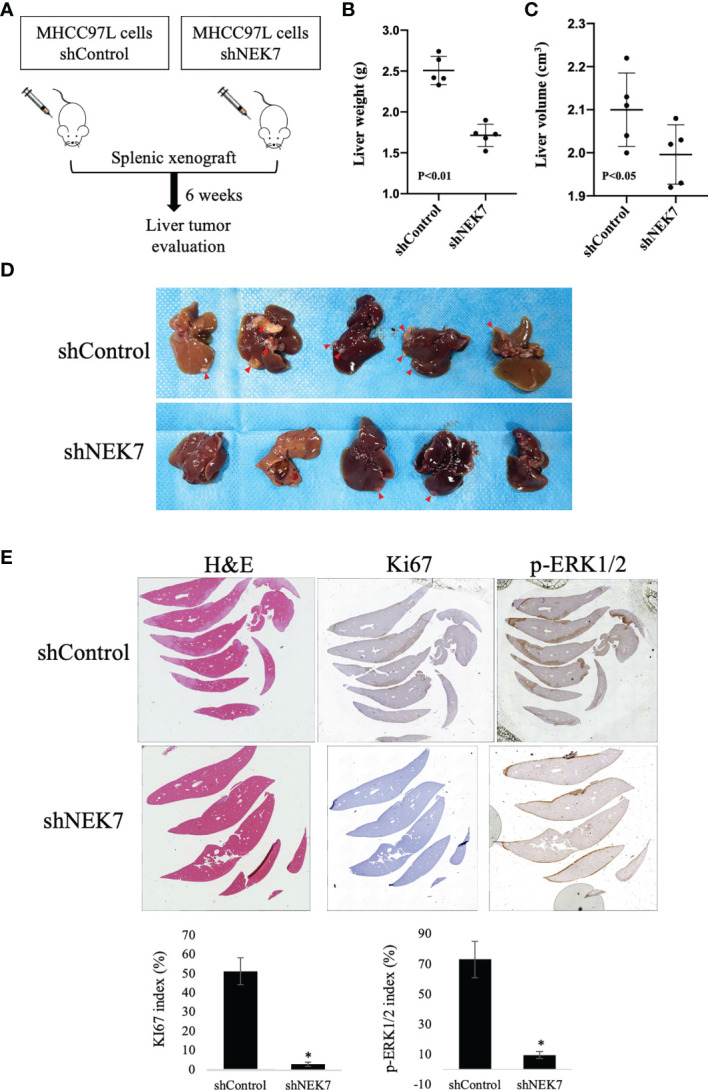
NEK7 decreased HCC tumor formation in a splenic xenograft mouse model. **(A)** Scheme of the xenograft experiment. Female nude mice were intrasplenic transplanted with MHCC97L cells and randomized divided into 2 groups (n = 5/group). 6 weeks after implantation, mice were sacrificed and liver tumors were harvested. **(B, C)** Downregulation of NEK7 decreased liver weight (*p* < 0.01) and volume (*p* < 0.05) of mice. **(D)** Gross pathology showed that knockdown of NEK7 significantly reduced liver metastasis formation (arrowheads: metastasis lesions). **(E)** H&E and IHC staining revealed decreased expression of Ki67 and p-ERK1/2 in livers of shNEK7 xenograft mice. *P < 0.05.

We next investigated the effect of NEK7 downregulation on HCC progression in a splenic xenograft mouse model. ShControl or shNEK7 MHCC97L cells were splenic transplanted into nude mice, and liver tumor growth was evaluated after 6 weeks of cell transplantation (**Figure 6A**). Compared to the control group, mice with NEK7-depleted cells exhibited decreased liver weight and volume (**Figures 6B–D**). Moreover, histological analysis revealed that cancer cells that colonized into the liver parenchyma were suppressed in mice injected with NEK7 knockdown cancer cells. Meanwhile, decreased expression of Ki67 and p-ERK1/2 indicates that knockdown of NEK7 suppressed the tumor formation capacity of HCCs (**Figure 6E**). Taken together, these results indicated that downregulation of NEK7 reduced the colonization and proliferation of HCCs and inhibited tumor formation *in vivo*.

## Discussion

In the present study, we examined a NEK7 expression pattern with GSDMD in l HCC and investigated the functional impact of NEK7-regulated pyroptosis on tumor progression. Our results showed that NEK7 expression was correlated with pyroptosis marker GSDMD. NEK7 was associated with the migratory, invasive, and proliferative capacities of HCCs. A coculture experiment indicated that NEK7-regulated cancer cell pyroptosis inhibited stimulation of HSC activation and tumor-stromal interaction of cancer cells. *In vivo* experiments using a splenic injection model revealed that downregulation of NEK7 inhibited HCC tumor formation. Taken together, these results suggest that NEK7-regulated pyroptosis may play an important role in HCC progression; targeting pyroptosis by inhibiting NEK7 could be an effective therapeutic strategy for HCC progression and cancer–stromal interaction.

Pyroptosis plays a dual role in the pathogenesis of tumors (36). On the one hand, the multiple signaling pathways and inflammatory chemokines released during pyroptosis are contributed to tumorigenesis and chemoresistance (37, 38). On the other hand, as a type of cell death, pyroptosis can inhibit the occurrence and development of tumors (37, 39). Wei et al. found a different result of the NLRP3 inflammasome in HCC: on the one hand, the NLPR3 expression was downregulated in HCC tissues compared to non-cancerous liver tissues, and its expression was negatively correlated with the pathological grade and clinical stage of HCC (19); on the other hand, in the comparison of normal liver tissue and liver cancer tissue, NLRP3 expression in normal liver tissue was low, while that in cancer tissues was upregulated. Furthermore, they found an anticancer effect of 17β-estradiol, by trigger pyroptosis *via* activation of the NLRP3 inflammasome (40). Another inflammasome signaling relevant gene, IRF2, was reported to regulate caspase-1 and GSDMD transcript expression in macrophages, endothelial cells, and multiple tissues (41). Moreover, LPS induces GSDMD expression change in varying degrees by mediating caspase-4 or caspase-11 (42, 43). An FDA-approved drug, disulfiram, protected against LPS-induced GSDMD pore formation and IL-1β leaking, while the expression of GSDMD was inhibited following treatment of disulfiram (44). Our data demonstrated a knockdown of NEK7 inhibited GSDMD expression, and this effect was strengthened in the presence of LPS and nigericin treatment. However, the regulation of GSDMD and cleaved-GSDMD is a complex process far from being clearly understood.

Cancer–stromal interactions reported a key regulator of HCC progression (32). Cancer cells are able to promote HSC activation and proliferation. Meanwhile, activated HSCs in turn generate a variety of cytokines, growth factors, and ECM proteins, which contribute to forming a microenvironment favorable for tumor growth and promoting tumor cell progression, metastasis, and chemotherapy resistance (33). Activated HSCs are myofibroblast-like cells that express α-smooth muscle actin (α-SMA) and become proliferative and contractile (45). By coculturing with HSCs, cancer cells enhanced proliferation and migration through ERK and NF-κB signaling pathways (34). Co-inoculation of activated primary HSCs with tumor cells *in vivo* leads to enhanced tumor growth and proliferation of HCC (46). Currently, therapeutic approaches are used to target tumor cells of l HCC; recent studies have instead focused on potentially targeting the stromal cell therapeutic approach to HCC treatment (47). We provide new insights into and perspectives for effective therapeutic HSC targeting, which exhibited a brand-new strategy through tumor cell pyroptosis.

In conclusion, NEK7 increased the cell migration and invasion capacity of HCC. Downregulation of NEK7 suppressed cancer–stromal interaction by inducing cancer cell pyroptosis. NEK7 promoted HCC cell growth *in vitro* and *in vivo*. Our findings suggest that NEK7 plays an important role in cancer–stromal interaction of HCC. Investigation of NEK7-regulated pyroptosis could move forward for revealing the genomic alteration of HCC. Moreover, anti-NEK7 drugs may have potential to regulate NLRP3 to abolish the inflammation response and NLRP3-related diseases (48, 49). Therefore, NEK7 targeting therapy might be a potential new strategy for HCC treatment.

## Data Availability Statement

The original contributions presented in the study are included in the article/**Supplementary Material**. Further inquiries can be directed to the corresponding authors.

## Ethics Statement

The research study was approved by the Ethics Committee of Human Experimentation at the Peking University Shenzhen Hospital (Shenzhen, China). All patients studied signed informed consent for participation. The patients/participants provided their written informed consent to participate in this study. The animal study was approved by the Animal Ethics Committee of Peking University Shenzhen Hospital. All animal procedures and care were conducted in accordance with institutional guidelines and in compliance with national and international laws and policies.

## Author Contributions

ZY designed the study, conducted experiments, acquired and analyzed data, and wrote the manuscript. QD, ZFL analyzed bioinformatic data. JY, YS, QL and ZWL discussed and revised the manuscript. WY, GY, XL and QR conducted evaluation of pathology study. JQ and JL was responsible for the conception and supervision of the study and wrote the manuscript. All authors corrected drafts and approved the final version of the manuscript.

## Funding

This study was supported by the Sanming Project of Medicine in Shenzhen (No. SZSM201612021) and China Postdoctoral Science Foundation (No. 2020M682841).

## Conflict of Interest

The authors declare that the research was conducted in the absence of any commercial or financial relationships that could be construed as a potential conflict of interest.

## Publisher’s Note

All claims expressed in this article are solely those of the authors and do not necessarily represent those of their affiliated organizations, or those of the publisher, the editors and the reviewers. Any product that may be evaluated in this article, or claim that may be made by its manufacturer, is not guaranteed or endorsed by the publisher.

## Supplementary Material

Supplementary Figure 1 Bioinformatics analysis of NEK7 in hepatocellular carcinoma. **(A)** Expression of NEK7 significantly correlated to liver hepatocellular carcinoma cancer histological subtypes. ****P* < 0.001, **P* < 0.05. **(B)** Correlation between NEK7 expression and NLRP3, IL-18, GSDMB, GSDMC expression in HCC tissues. **(C)** Co-expression of NEK7 (green) and GSDMD (red) by multiple fluorescent immunohistochemical staining of HCC specimen. Scale bars = 100 μm.

Supplementary Figure 2 co-expression of NEK7 and GSDMD in HCC specimens. **(A)** mIHC staining of NEK7 and GSDMD on HCC specimens. **(B)** Co-expression of NEK7 and GSDMD was observed on serial sections of HCC specimens.

## Abbreviations

HCC, hepatocellular carcinoma; NEK7, NIMA-related protein kinase 7; NLRP3, NLR family pyrin domain containing 3; HSC, hepatic stellate cell; mIHC, multiplexed immunohistochemistry; LDH, lactate dehydrogenase; IL-1β, interleukin 1β; IL-18, interleukin 18; LPS, lipopolysaccharide; IHC, immunohistochemical; qRT-PCR, real-time quantitative polymerase chain reaction; FBS, fetal bovine serum; NC, negative control; shRNA, short hairpin RNA; TCGA, Cancer Genome Atlas Program; GSDMD, Gasdermin D; α-SMA, α-smooth muscle actin.
