# Supplementary figures and images for: Inhibition of NEK7 Suppressed Hepatocellular Carcinoma Progression by Mediating Cancer Cell Pyroptosis
Source: Front Oncol. 2022 Feb 10;12:812655. doi: 10.3389/fonc.2022.812655 (PMC8866693; doi:10.3389/fonc.2022.812655)

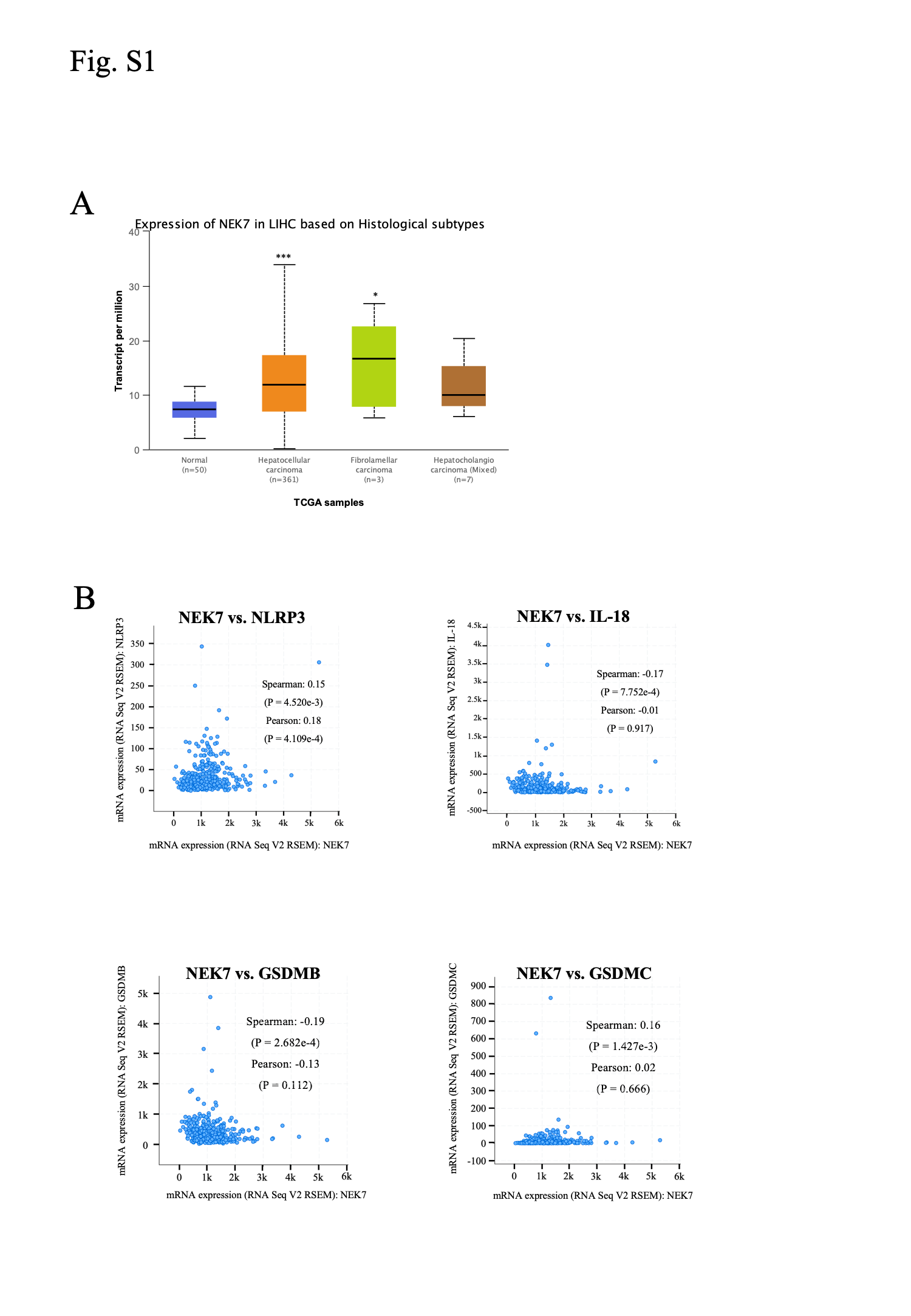

Supplement: Supplementary Figure 1 — Bioinformatics analysis of NEK7 in hepatocellular carcinoma. (A) Expression of NEK7 significantly correlated to liver hepatocellular carcinoma cancer histological subtypes. ***P < 0.001, *P < 0.05. (B) Correlation between NEK7 expression and NLRP3, IL-18, GSDMB, GSDMC expression in HCC tissues. (C) Co-expression of NEK7 (green) and GSDMD (red) by multiple fluorescent immunohistochemical staining of HCC specimen. Scale bars = 100 μm. [file Image_1.tiff]

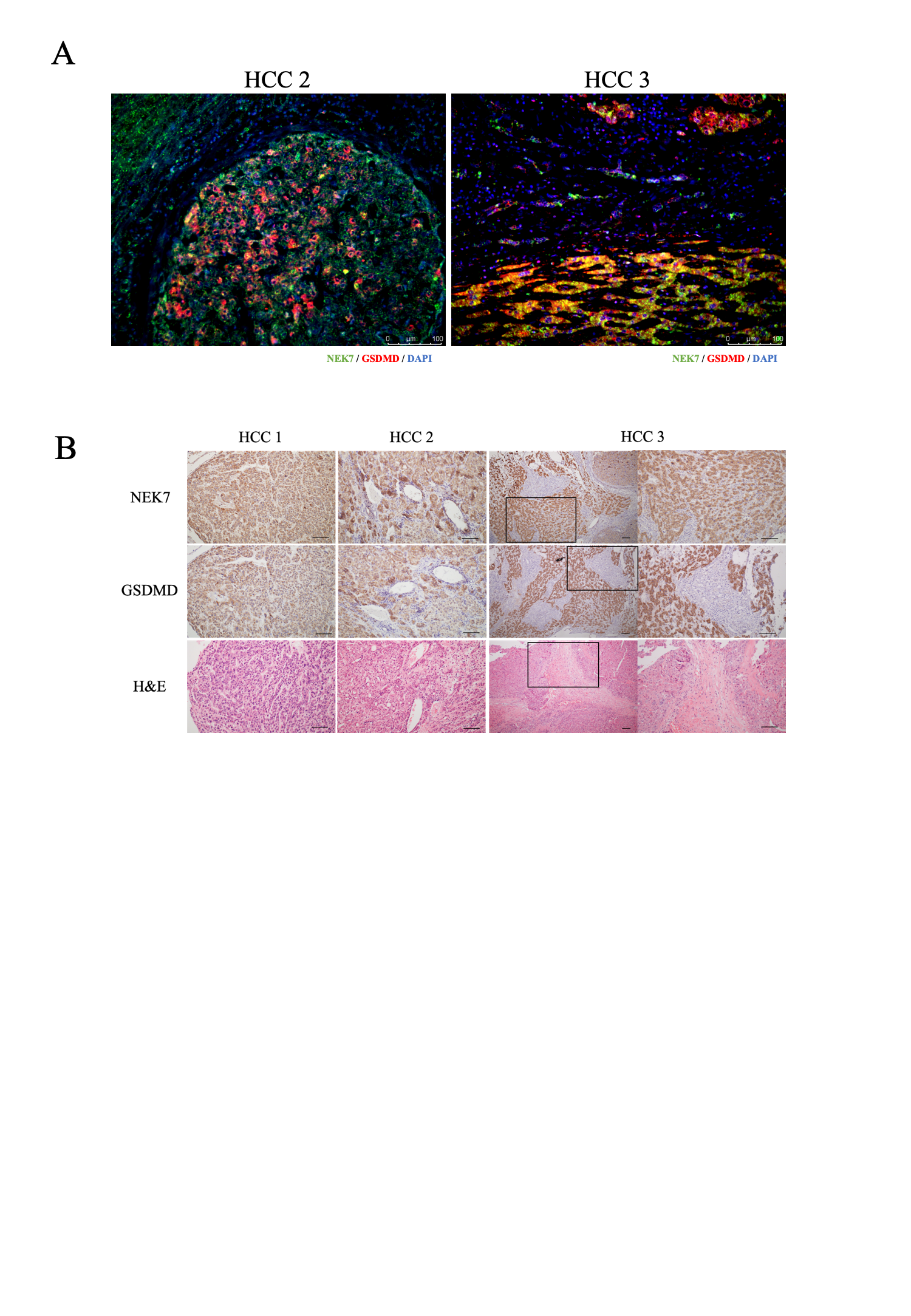

Supplement: Supplementary Figure 2 — co-expression of NEK7 and GSDMD in HCC specimens. (A) mIHC staining of NEK7 and GSDMD on HCC specimens. (B) Co-expression of NEK7 and GSDMD was observed on serial sections of HCC specimens. [file Image_2.tiff]

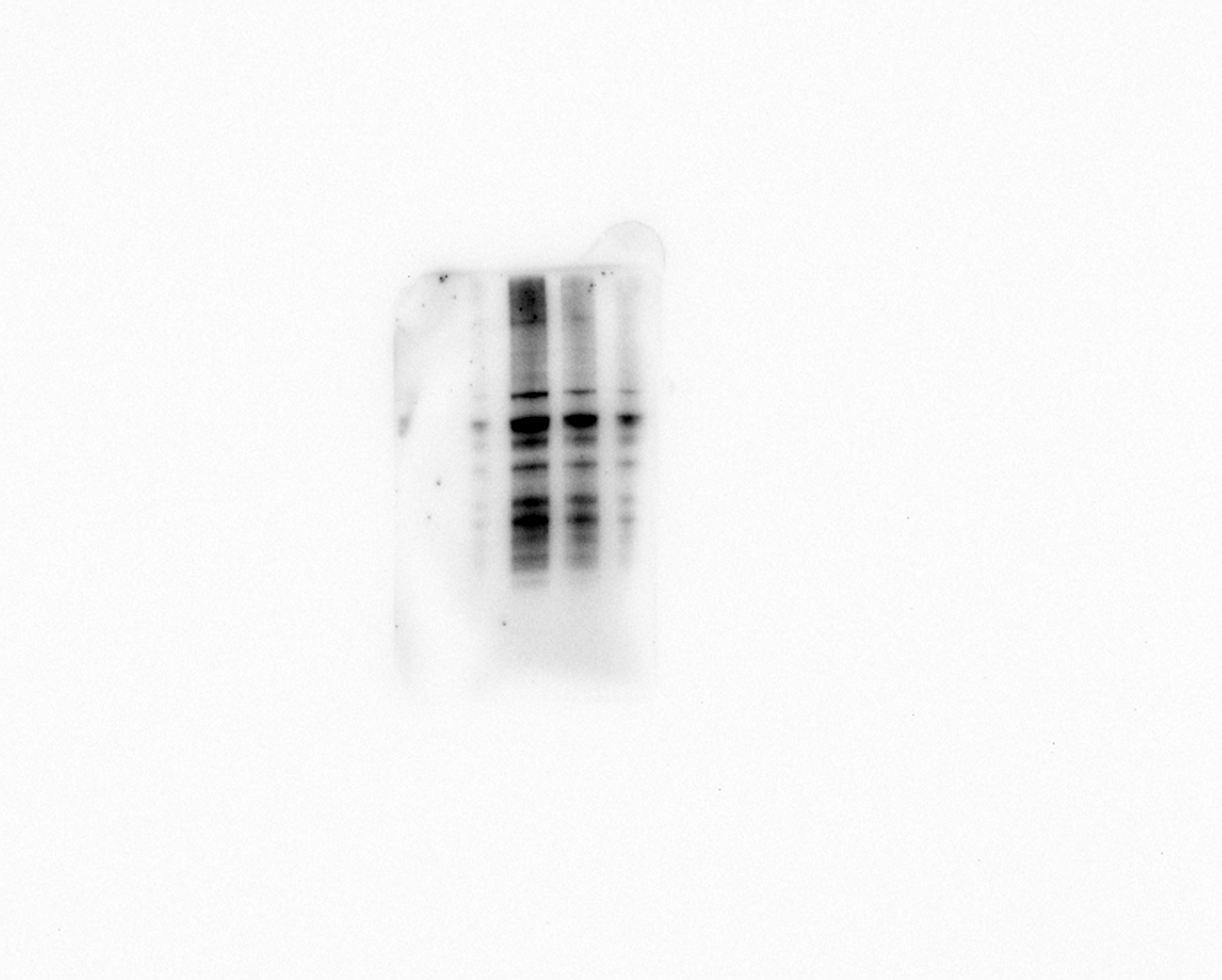

Supplement: Supplementary file 3 [file DataSheet_1.zip › Raw data of WB/Figure 4/Figure4- MHCC97L- GSDMD.Tif]

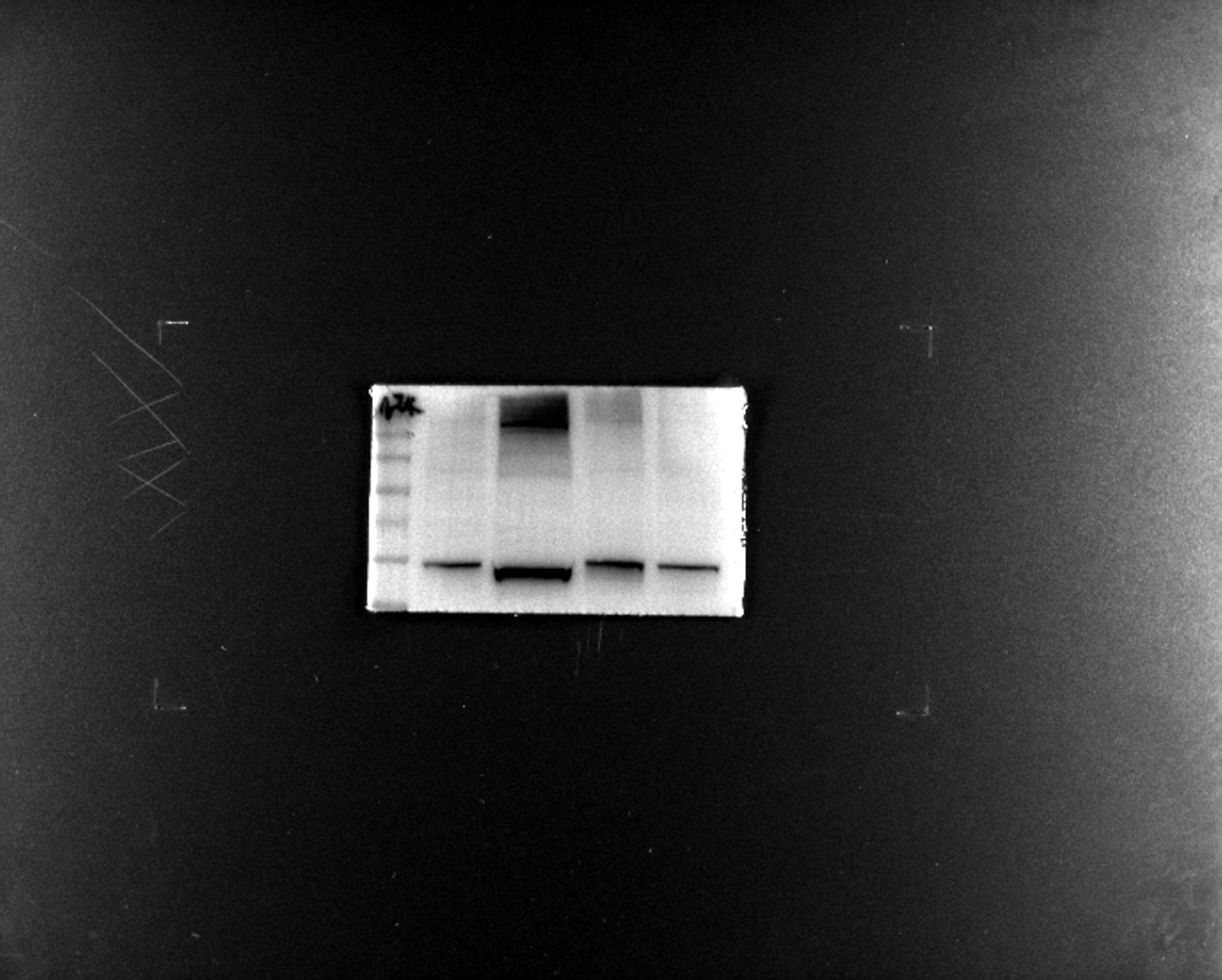

Supplement: Supplementary file 3 [file DataSheet_1.zip › Raw data of WB/Figure 4/Figure4- HepG2- GSDMD.Tif]

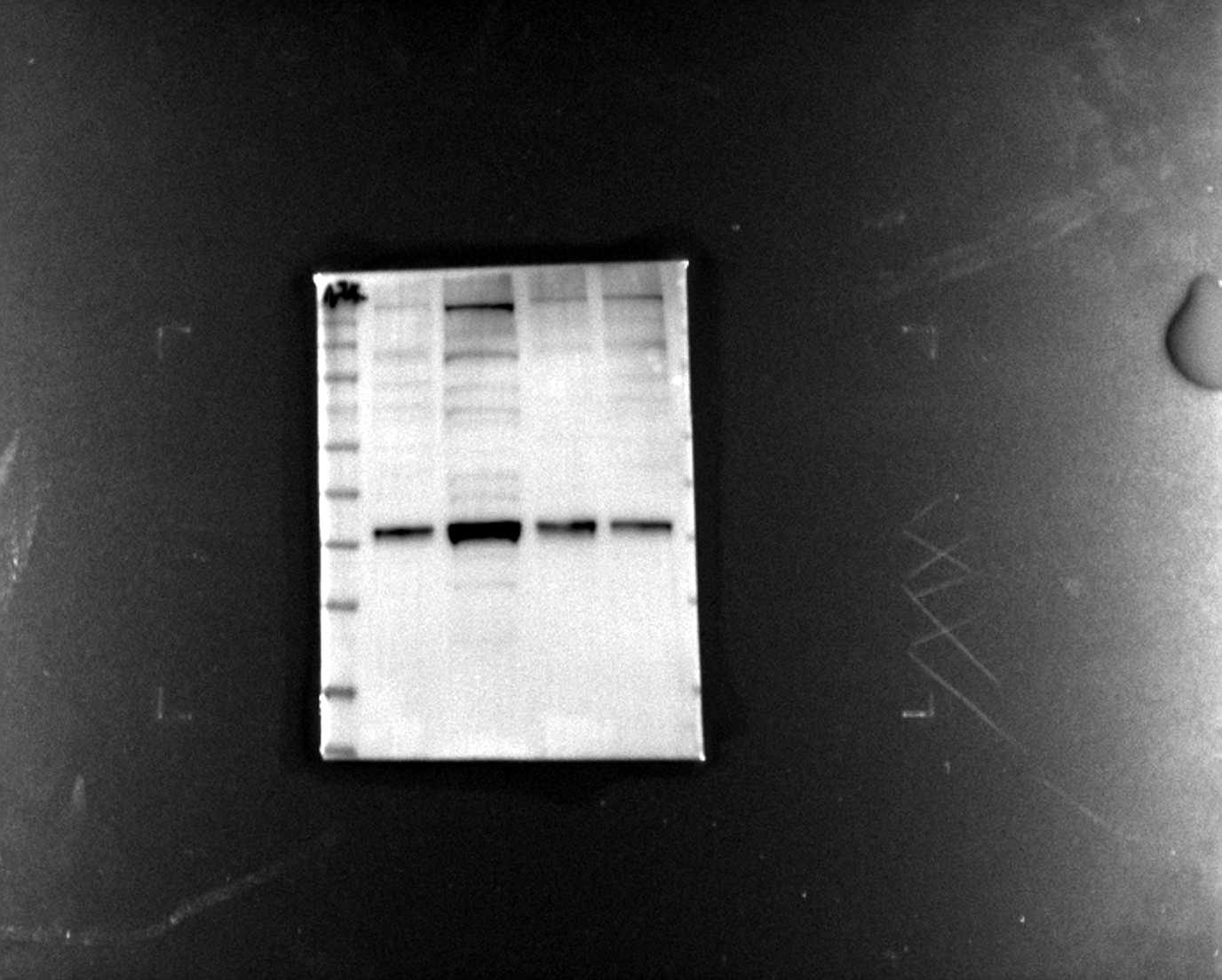

Supplement: Supplementary file 3 [file DataSheet_1.zip › Raw data of WB/Figure 4/Figure4- HepG2- NLRP3.Tif]

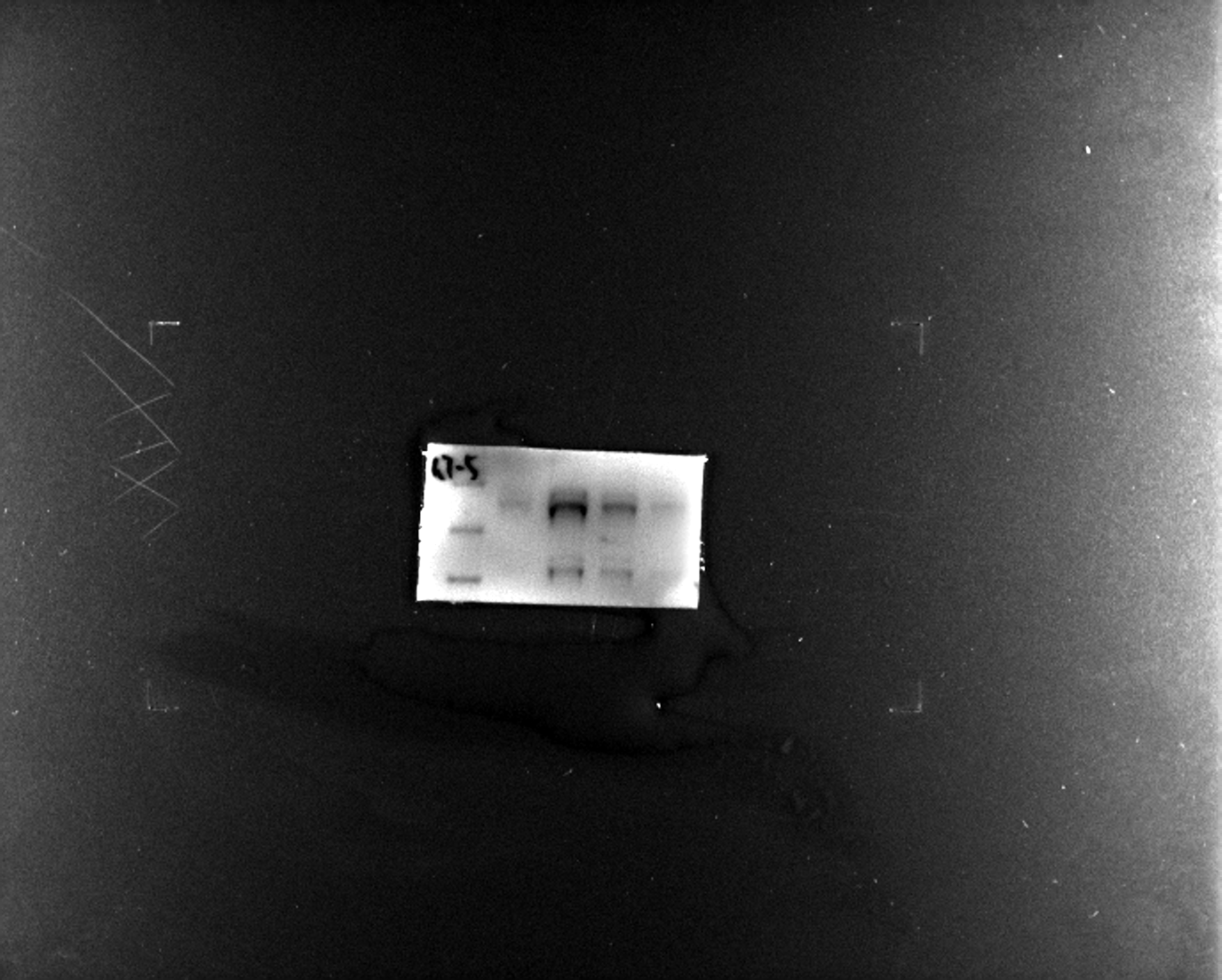

Supplement: Supplementary file 3 [file DataSheet_1.zip › Raw data of WB/Figure 4/Figure4- MHCC97L- NLRP3.Tif]

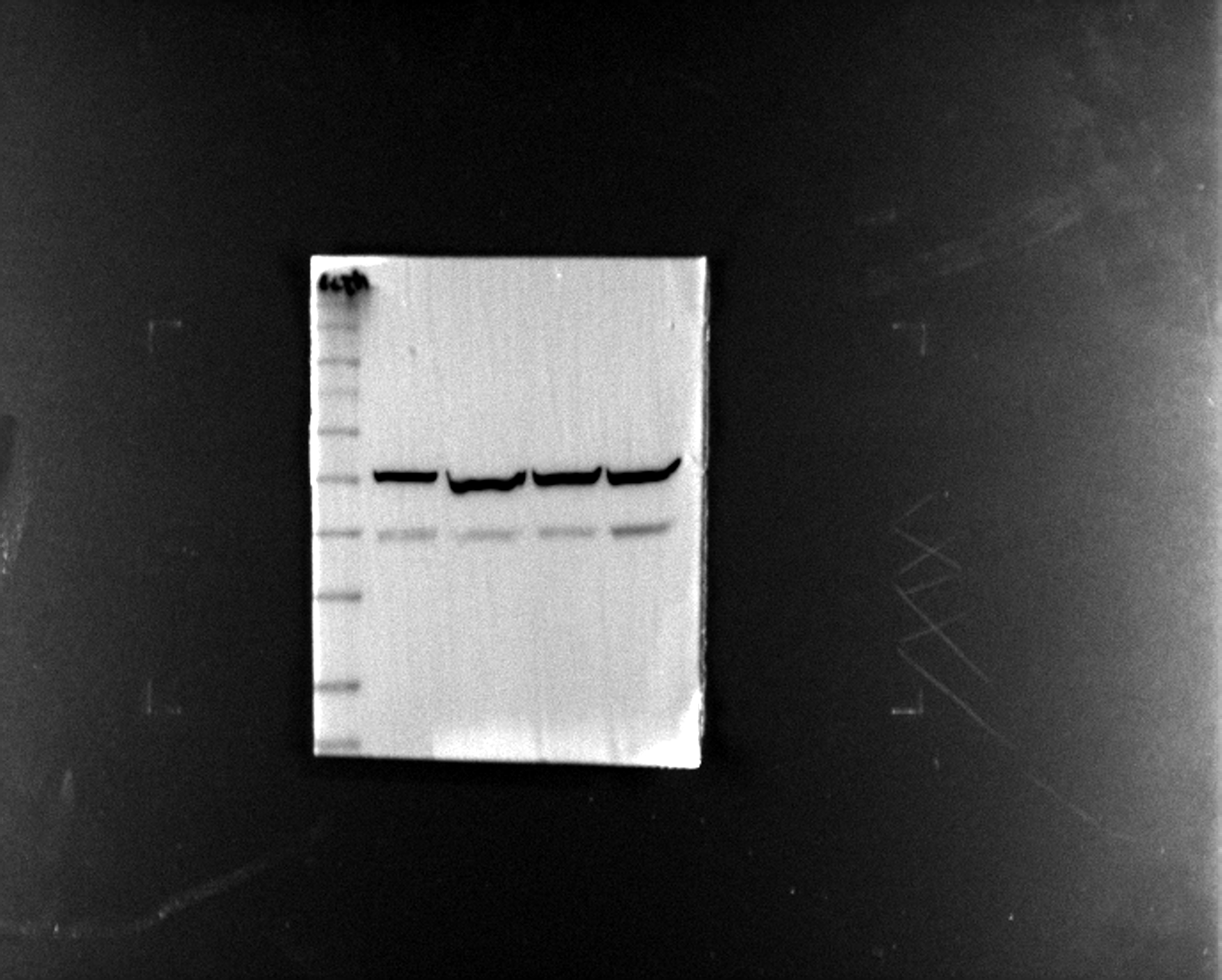

Supplement: Supplementary file 3 [file DataSheet_1.zip › Raw data of WB/Figure 4/Figure4- HepG2- ╬▓-actin.Tif]

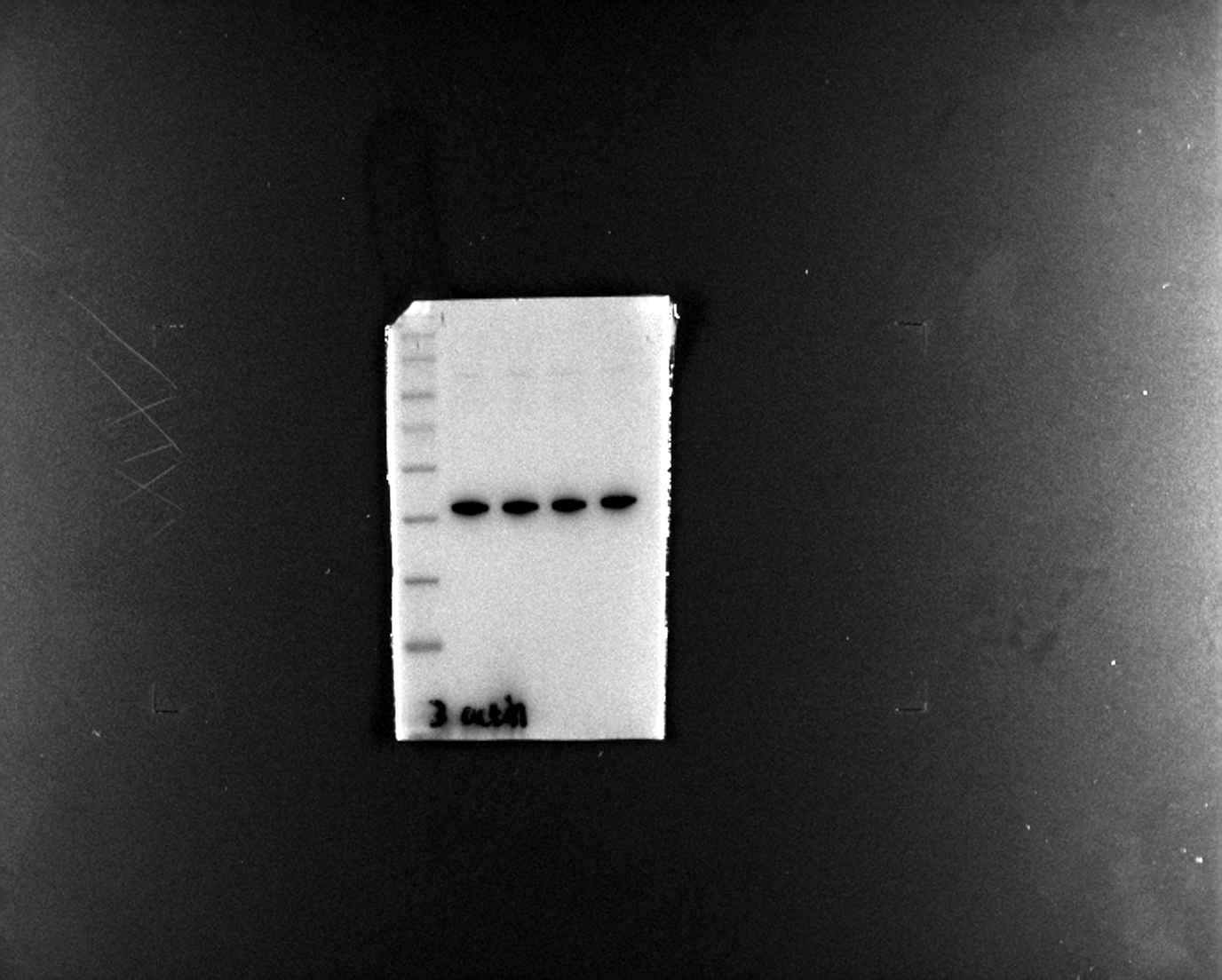

Supplement: Supplementary file 3 [file DataSheet_1.zip › Raw data of WB/Figure 4/Figure4- MHCC97L- ╬▓-ACTIN.Tif]

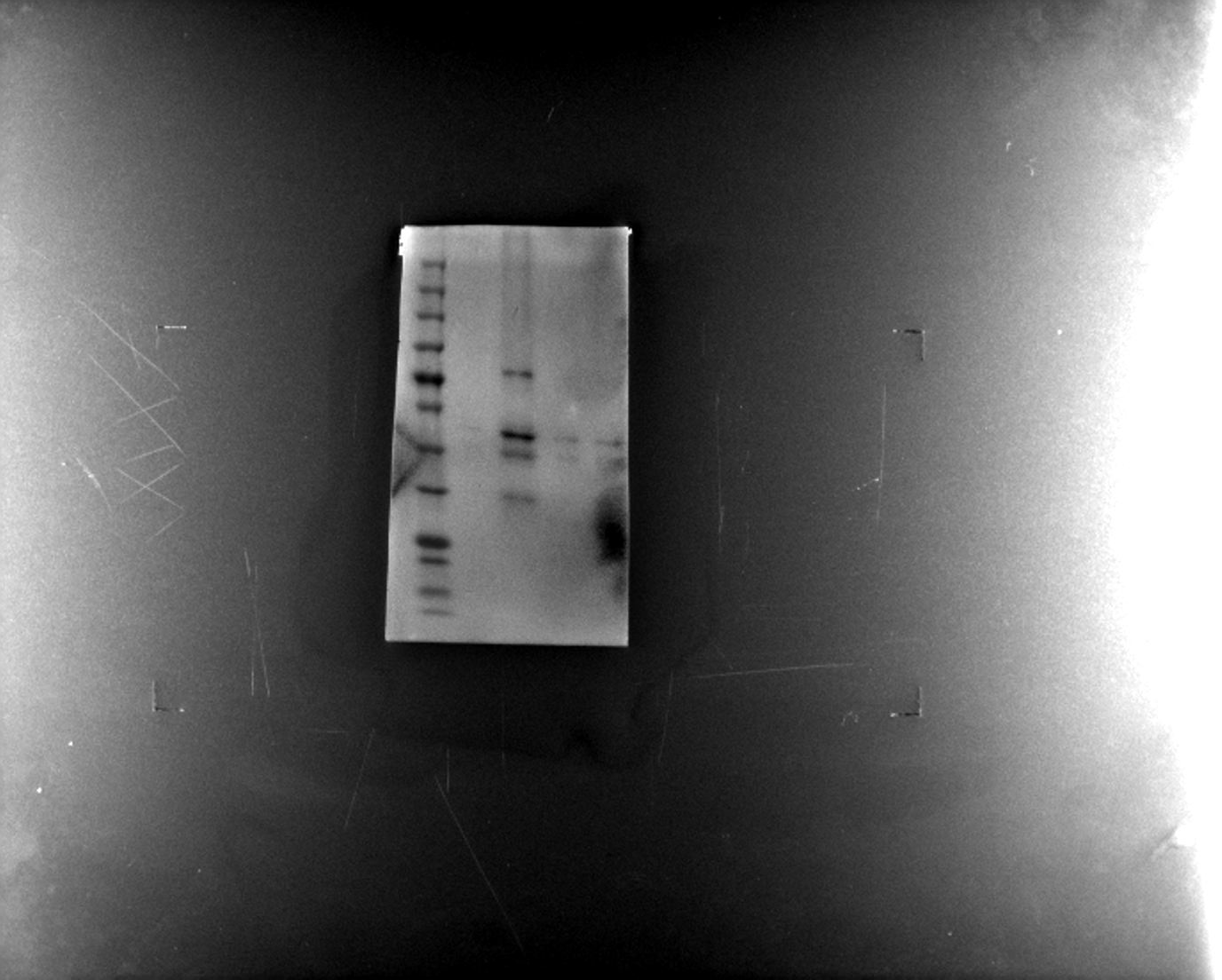

Supplement: Supplementary file 3 [file DataSheet_1.zip › Raw data of WB/Figure 4/Figure4- MHCC97L- Caspase1.Tif]

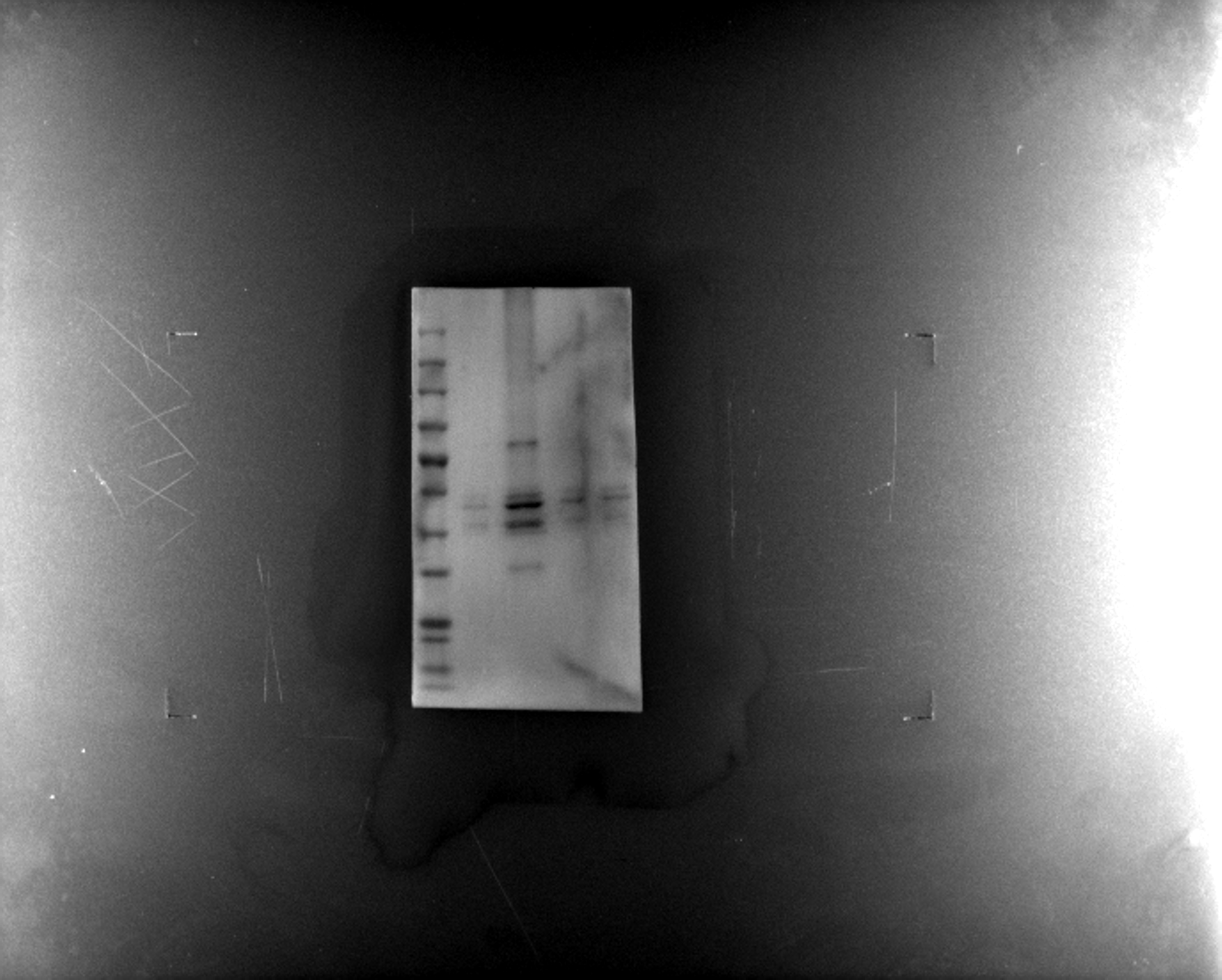

Supplement: Supplementary file 3 [file DataSheet_1.zip › Raw data of WB/Figure 4/Figure4- HepG2- Caspase1.Tif]

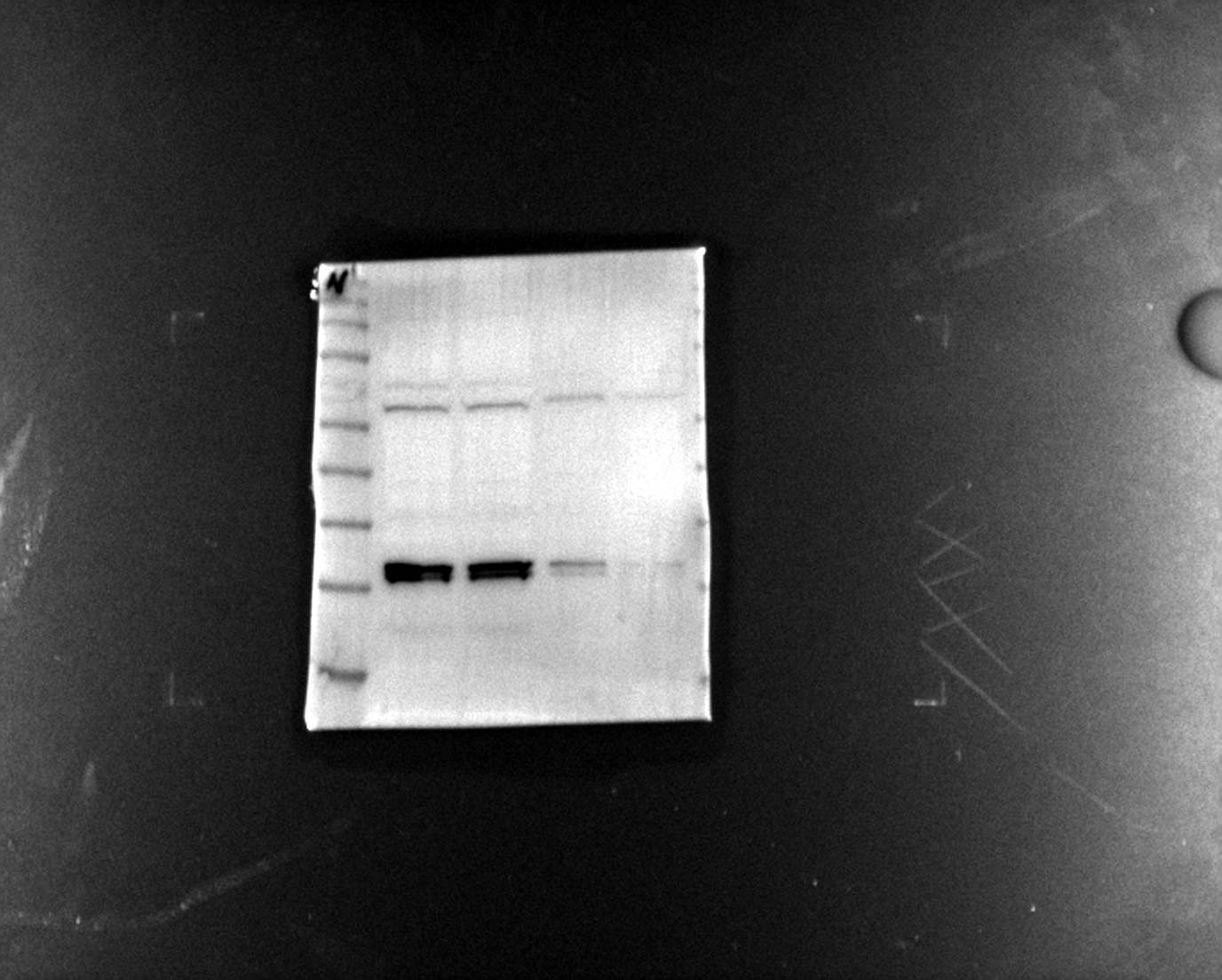

Supplement: Supplementary file 3 [file DataSheet_1.zip › Raw data of WB/Figure 4/Figure4- HepG2- NEK7.Tif]

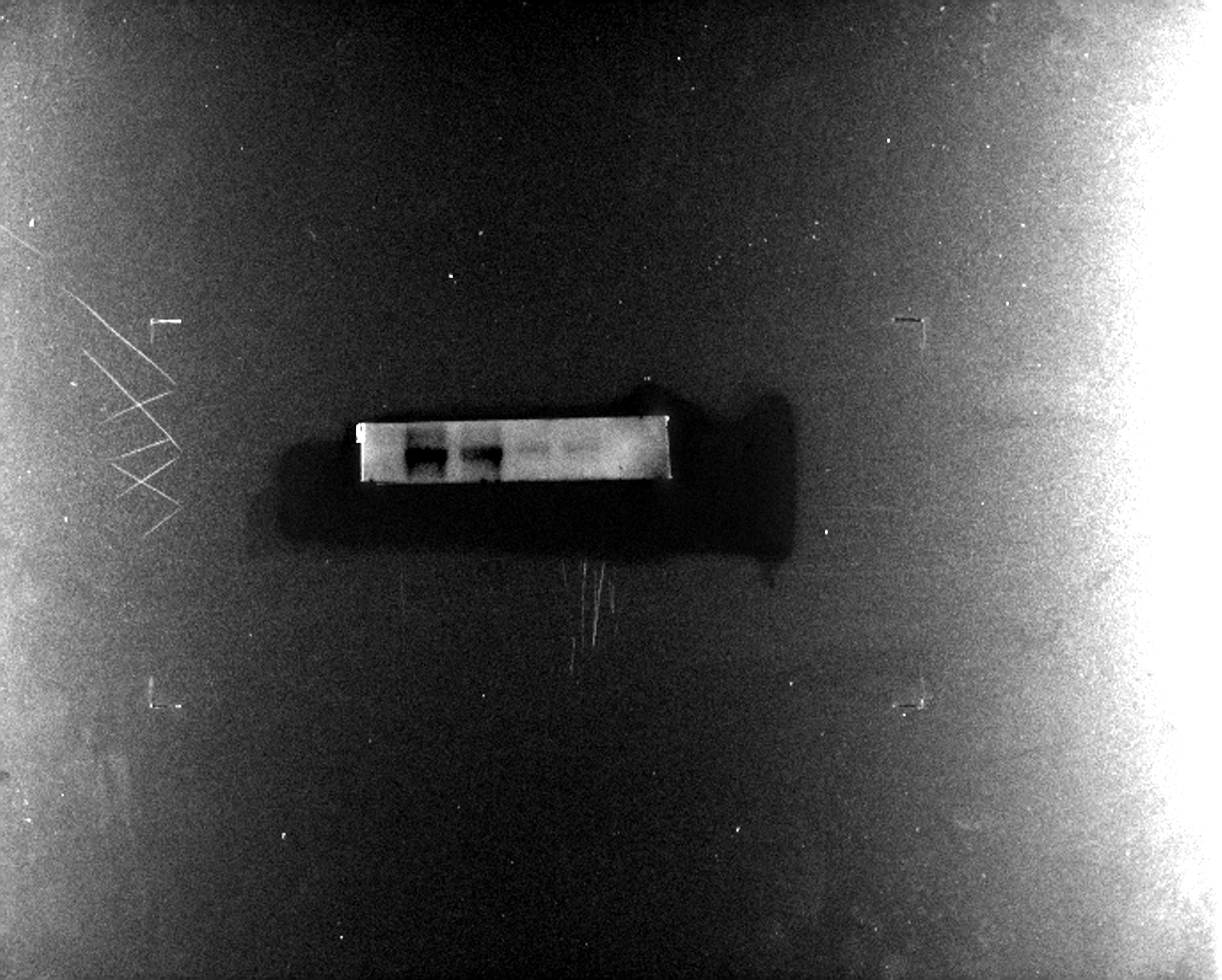

Supplement: Supplementary file 3 [file DataSheet_1.zip › Raw data of WB/Figure 4/Figure4- MHCC97L- NEK7.Tif]

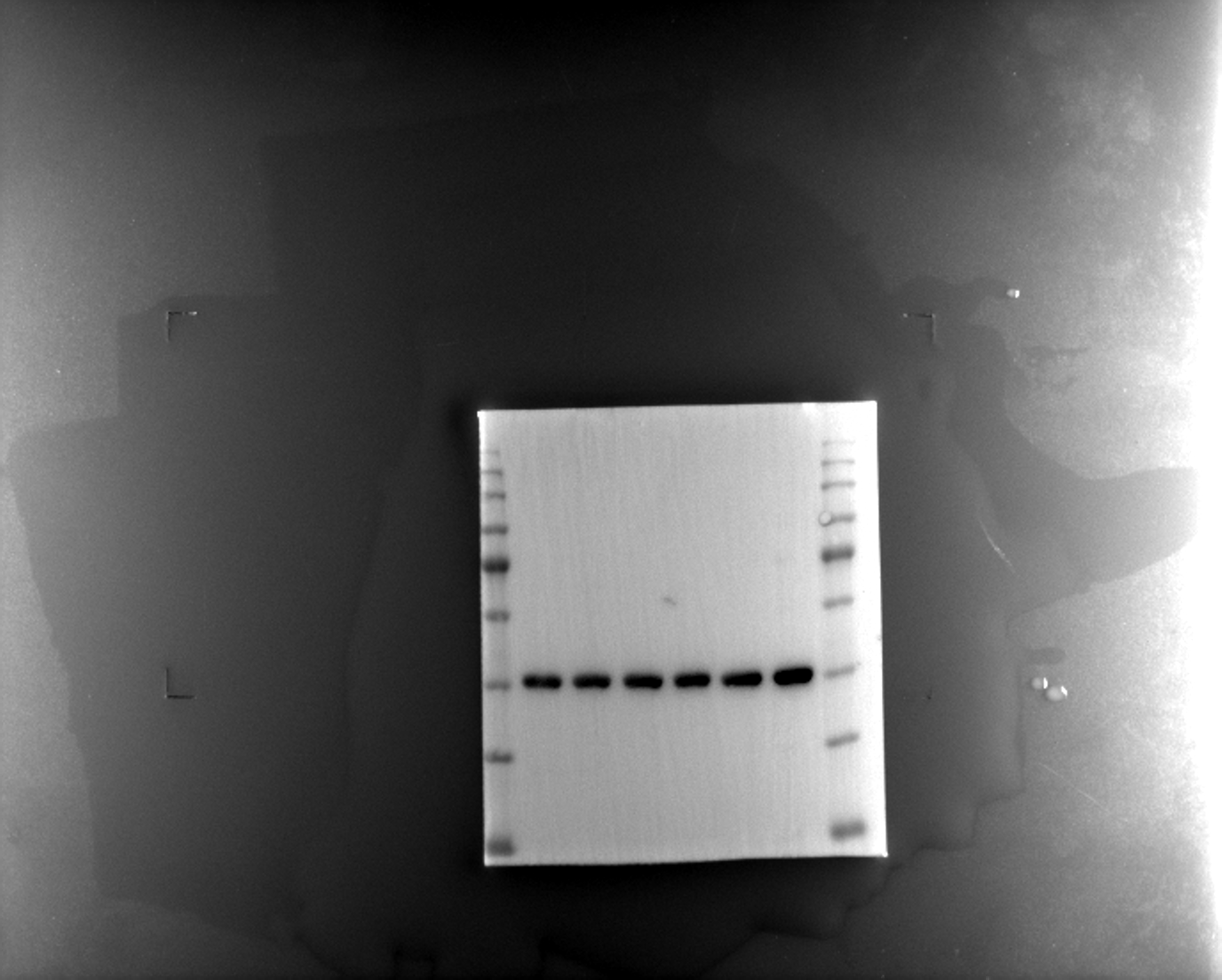

Supplement: Supplementary file 3 [file DataSheet_1.zip › Raw data of WB/Figure 3/Figure3- ╬▓-actin.Tif]

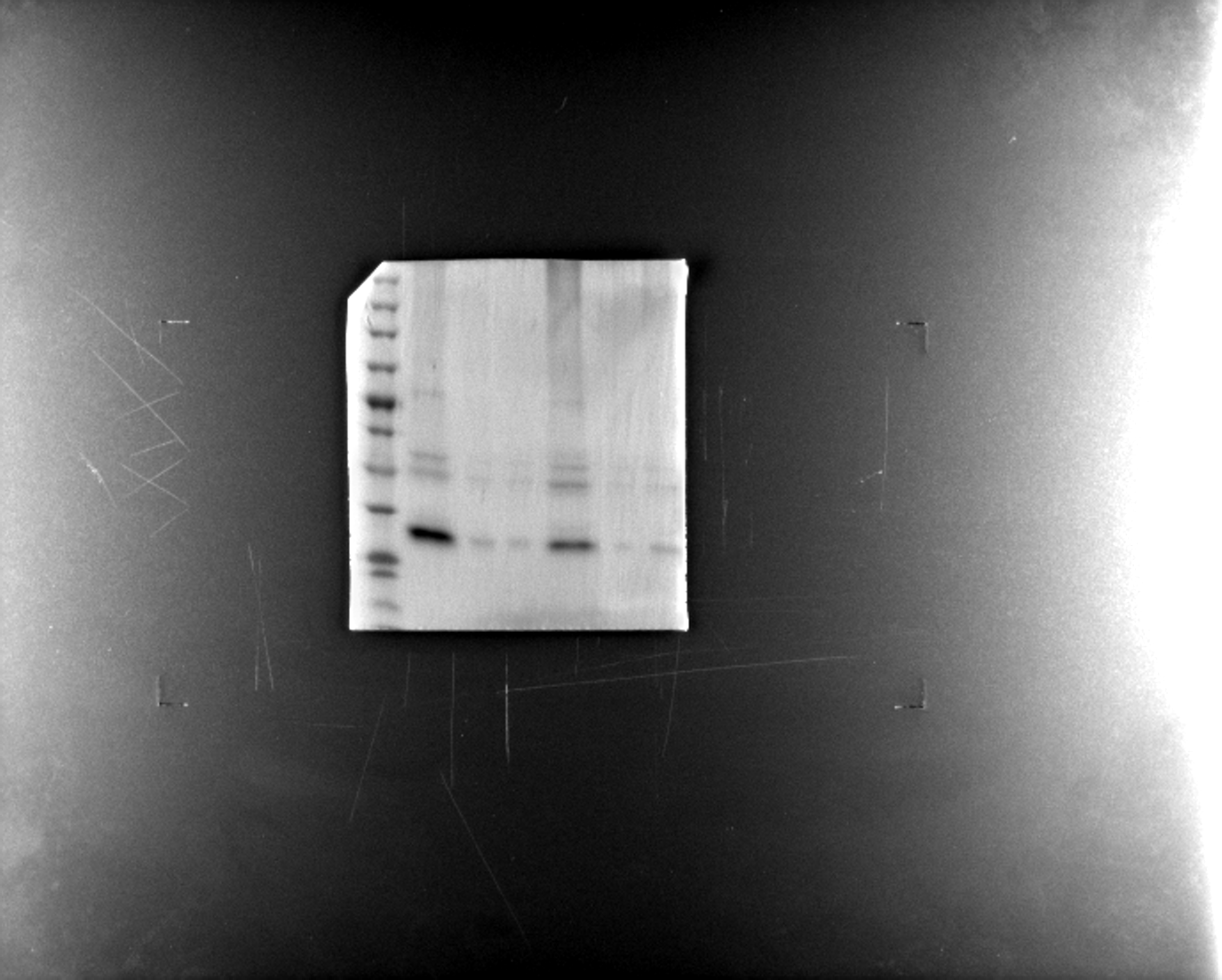

Supplement: Supplementary file 3 [file DataSheet_1.zip › Raw data of WB/Figure 3/Figure3- NEK7.Tif]

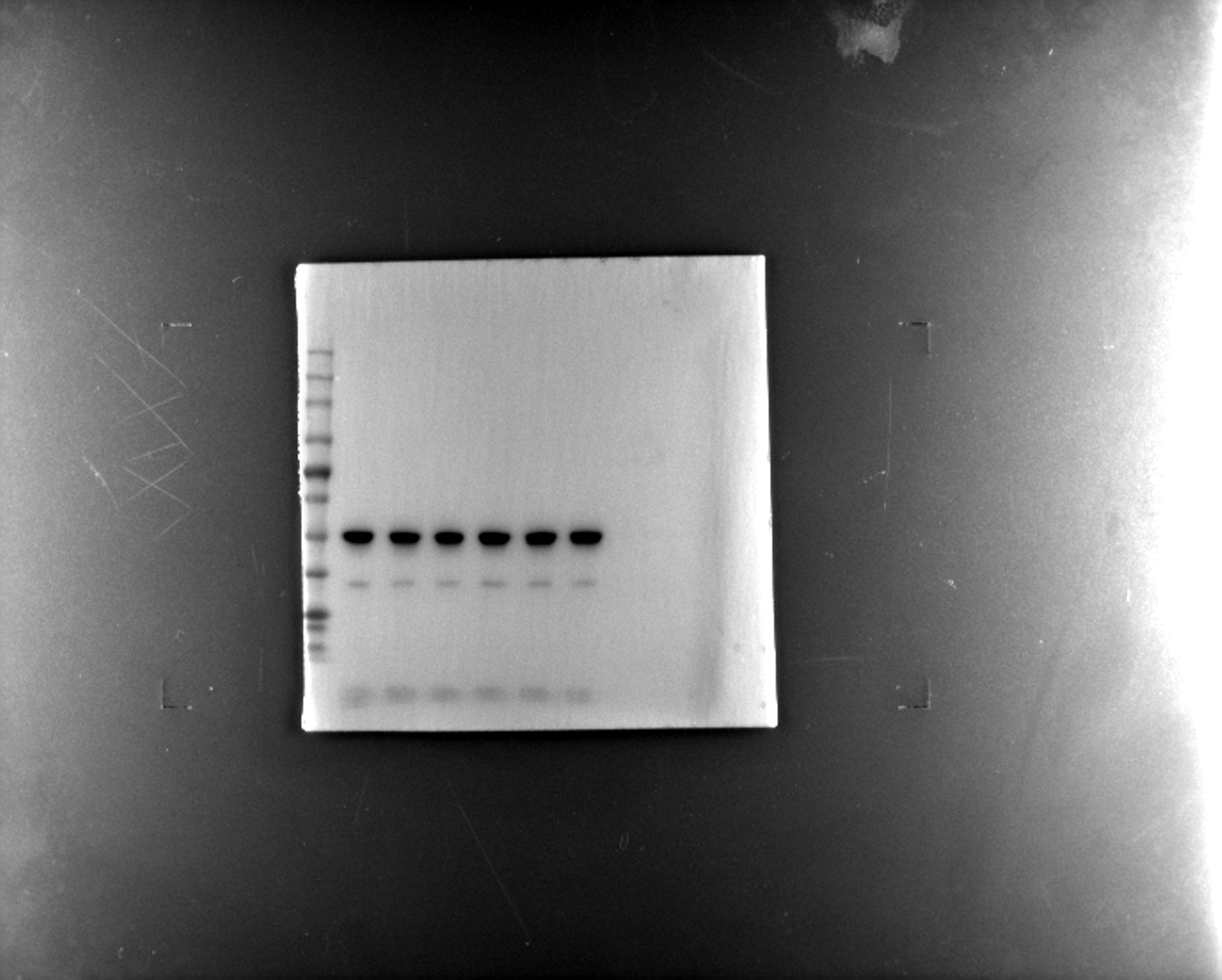

Supplement: Supplementary file 3 [file DataSheet_1.zip › Raw data of WB/Figure 2/Figure2- actin.Tif]

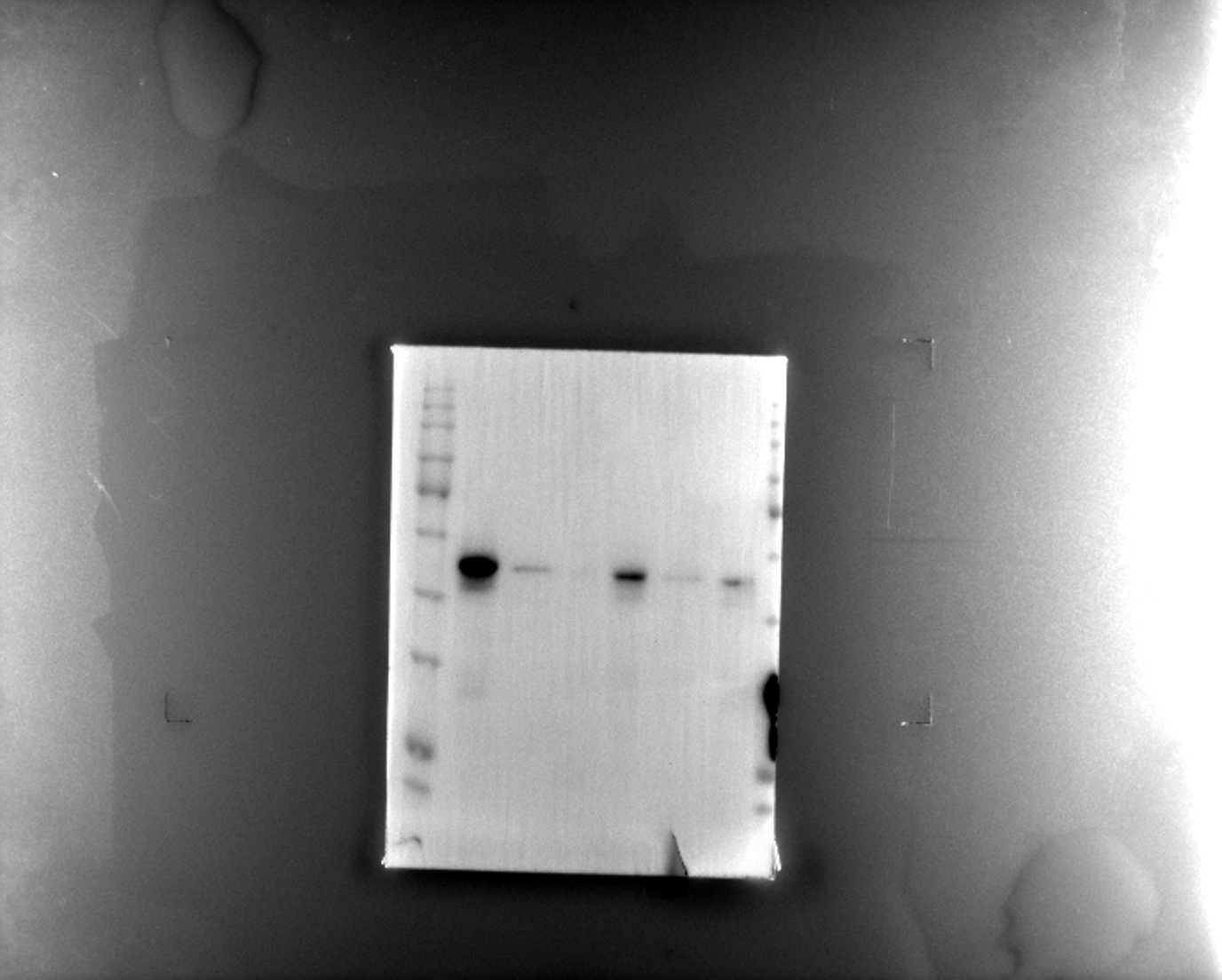

Supplement: Supplementary file 3 [file DataSheet_1.zip › Raw data of WB/Figure 2/Figure2- GSDMD.Tif]

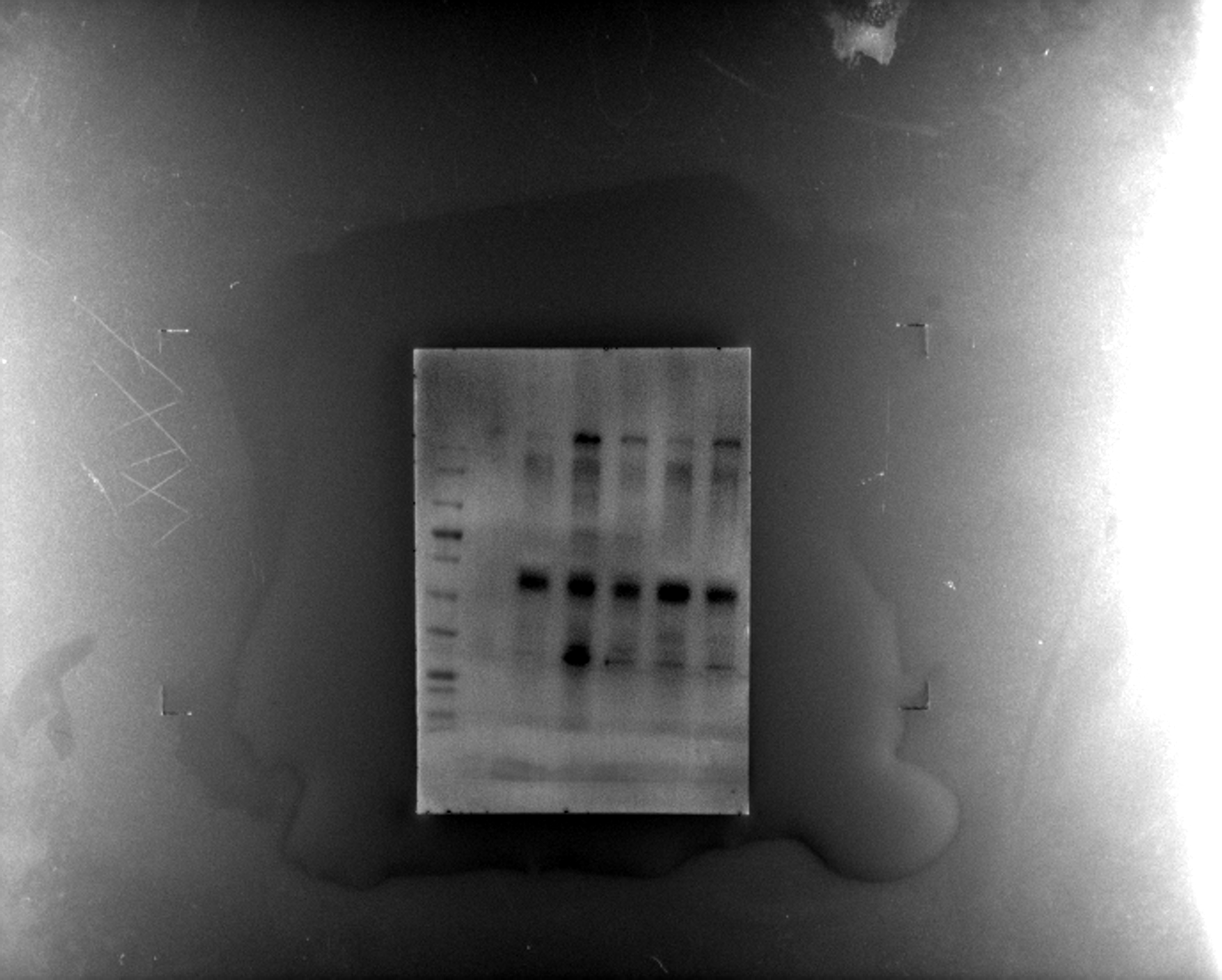

Supplement: Supplementary file 3 [file DataSheet_1.zip › Raw data of WB/Figure 2/Figure2- NEK7.Tif]

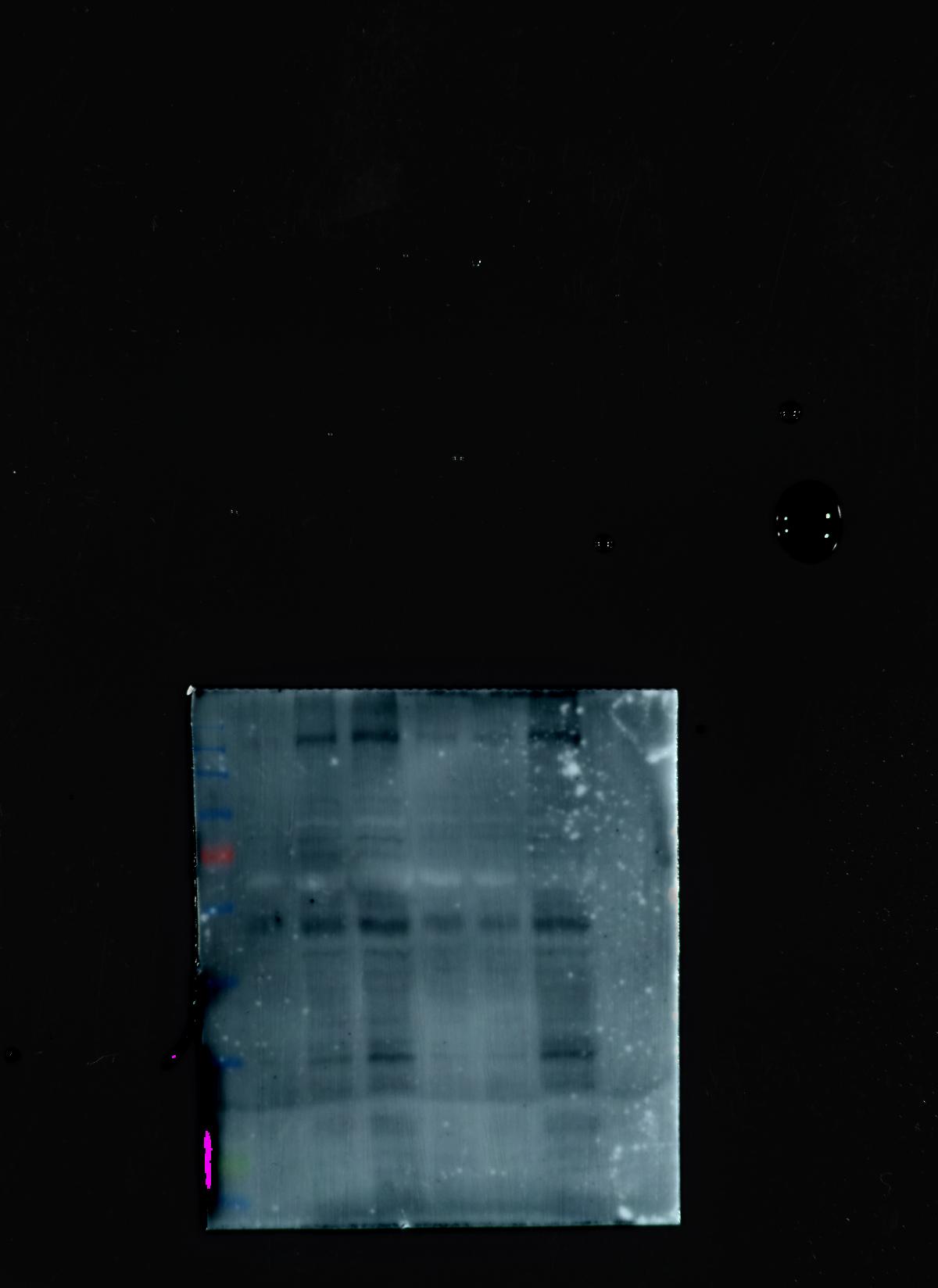

Supplement: Supplementary file 3 [file DataSheet_1.zip › Raw data of WB/Figure 2/Figure2- NLRP3.jpg]

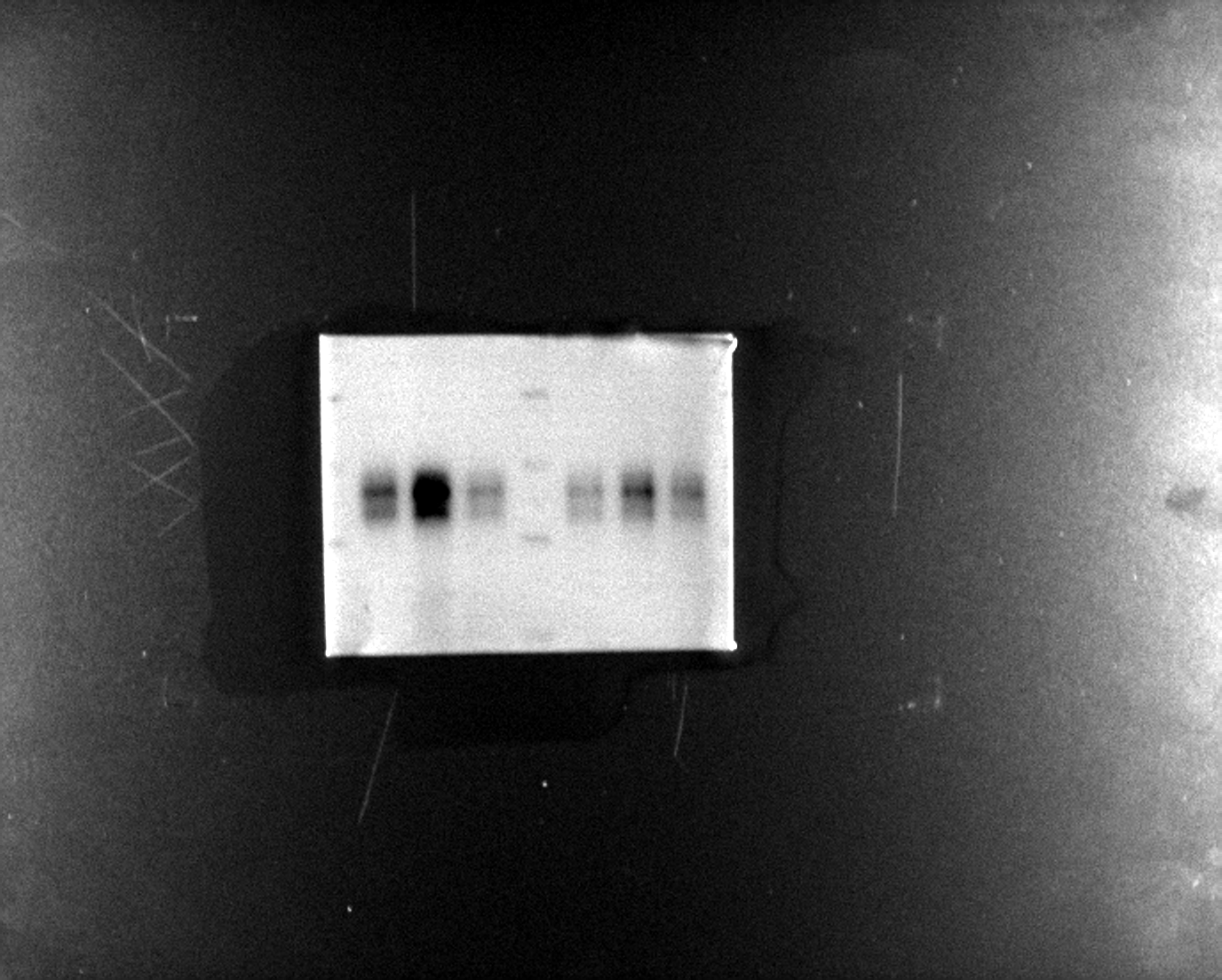

Supplement: Supplementary file 3 [file DataSheet_1.zip › Raw data of WB/Figure 5/Figure5- ╬▒-SMA.Tif]

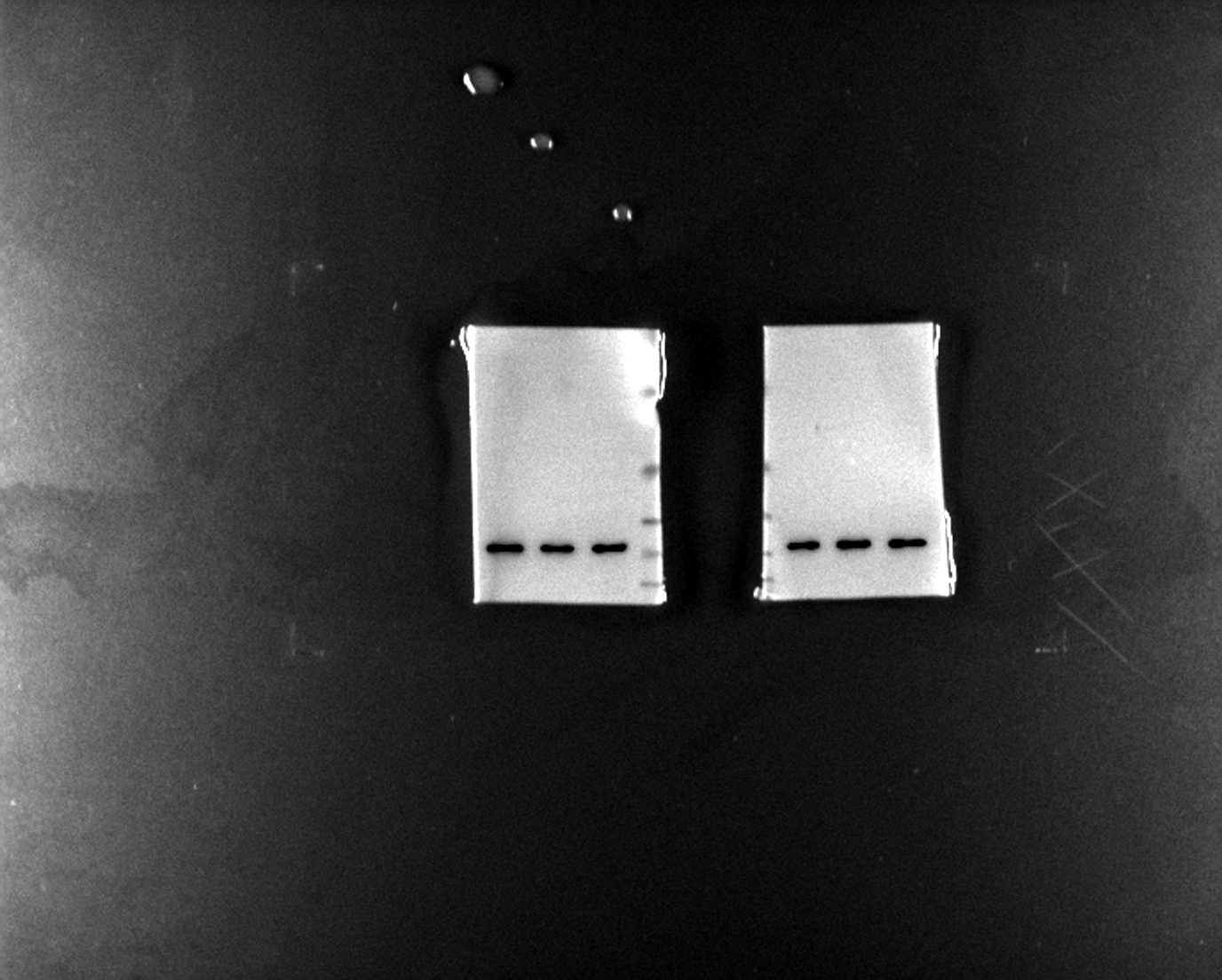

Supplement: Supplementary file 3 [file DataSheet_1.zip › Raw data of WB/Figure 5/Figure5- actin.Tif]

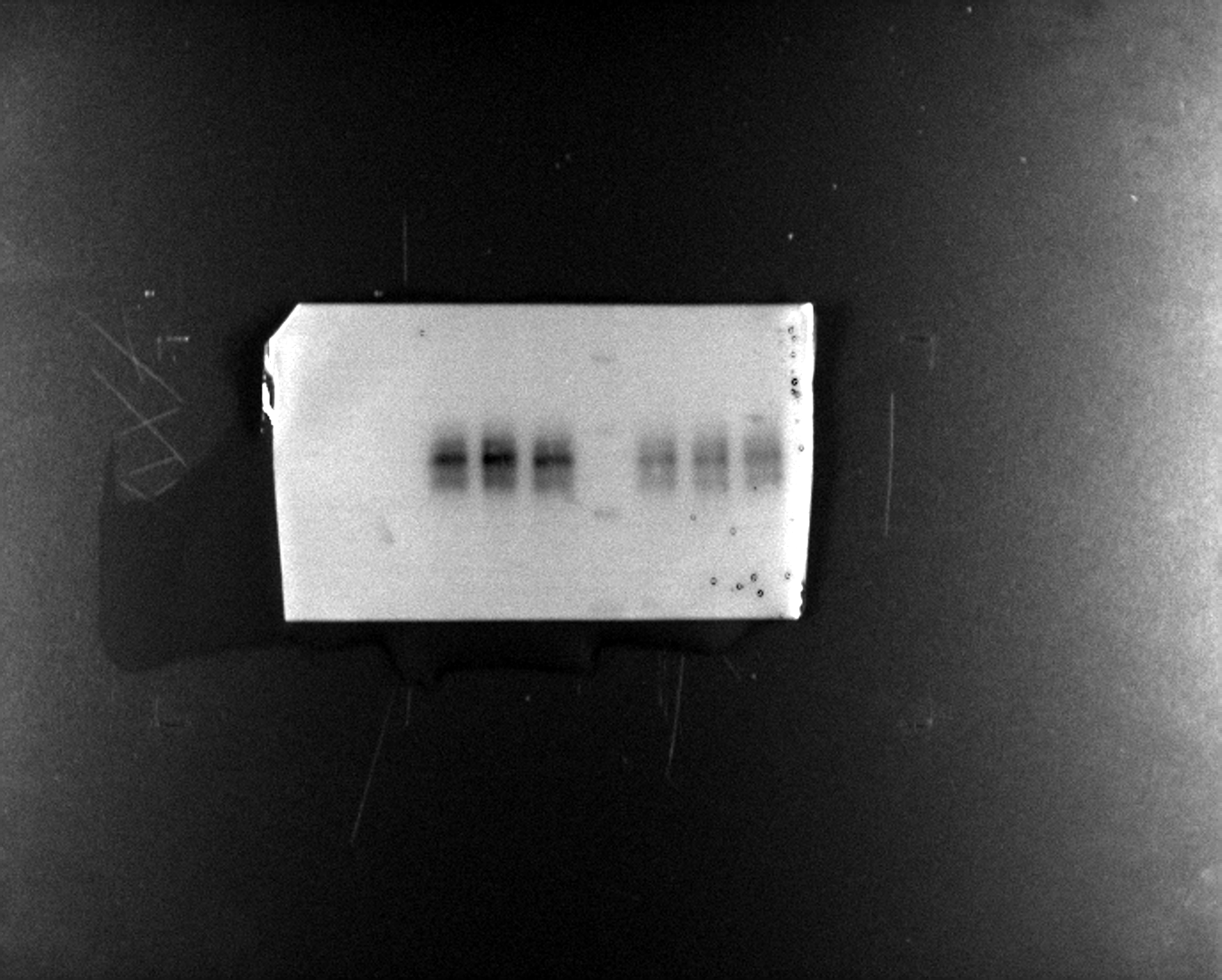

Supplement: Supplementary file 3 [file DataSheet_1.zip › Raw data of WB/Figure 5/Figure5- ERK1:2.Tif]

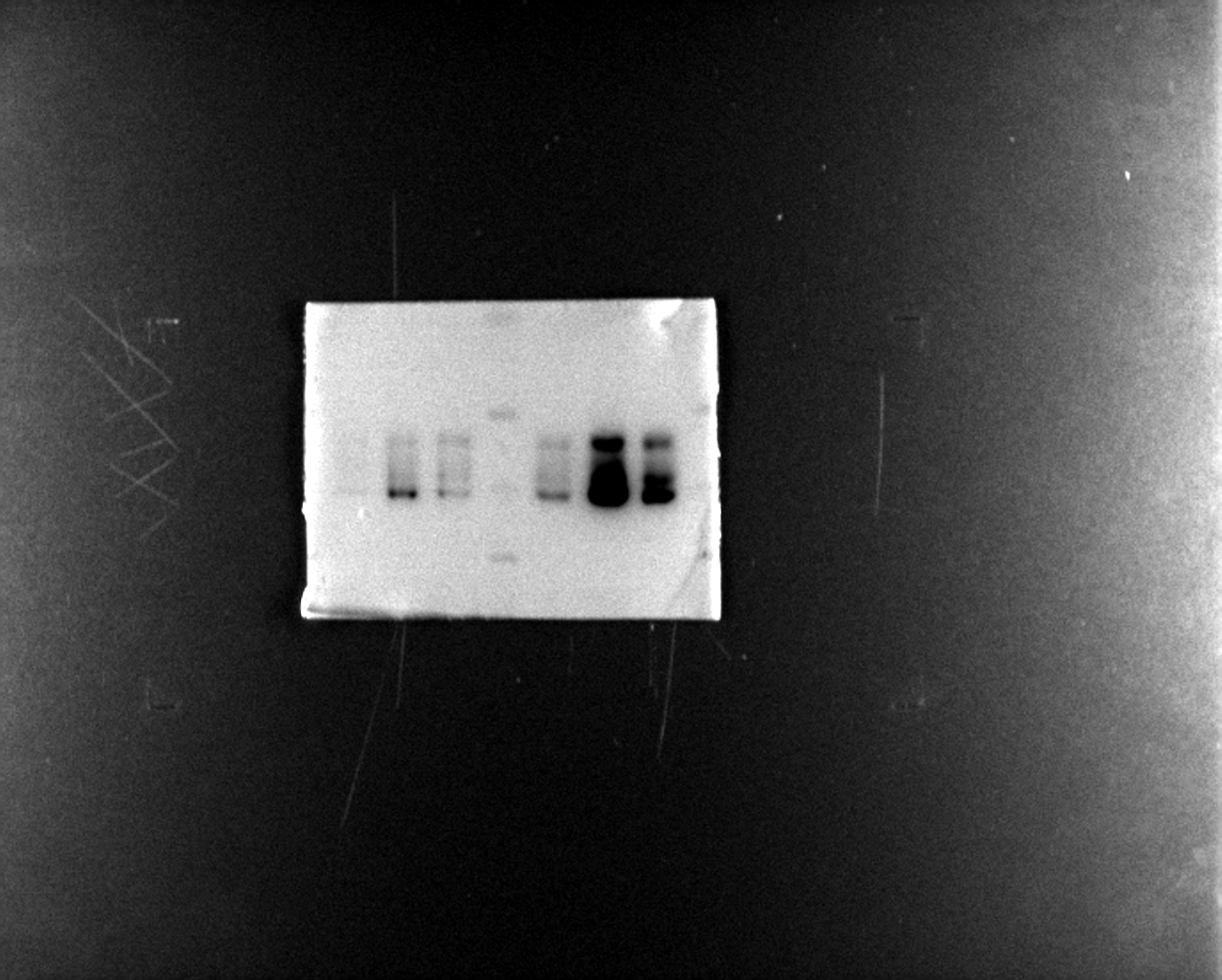

Supplement: Supplementary file 3 [file DataSheet_1.zip › Raw data of WB/Figure 5/Figure5- p-ERK1:2.Tif]

# Expression of GSDMD in LIHC based on Sample types

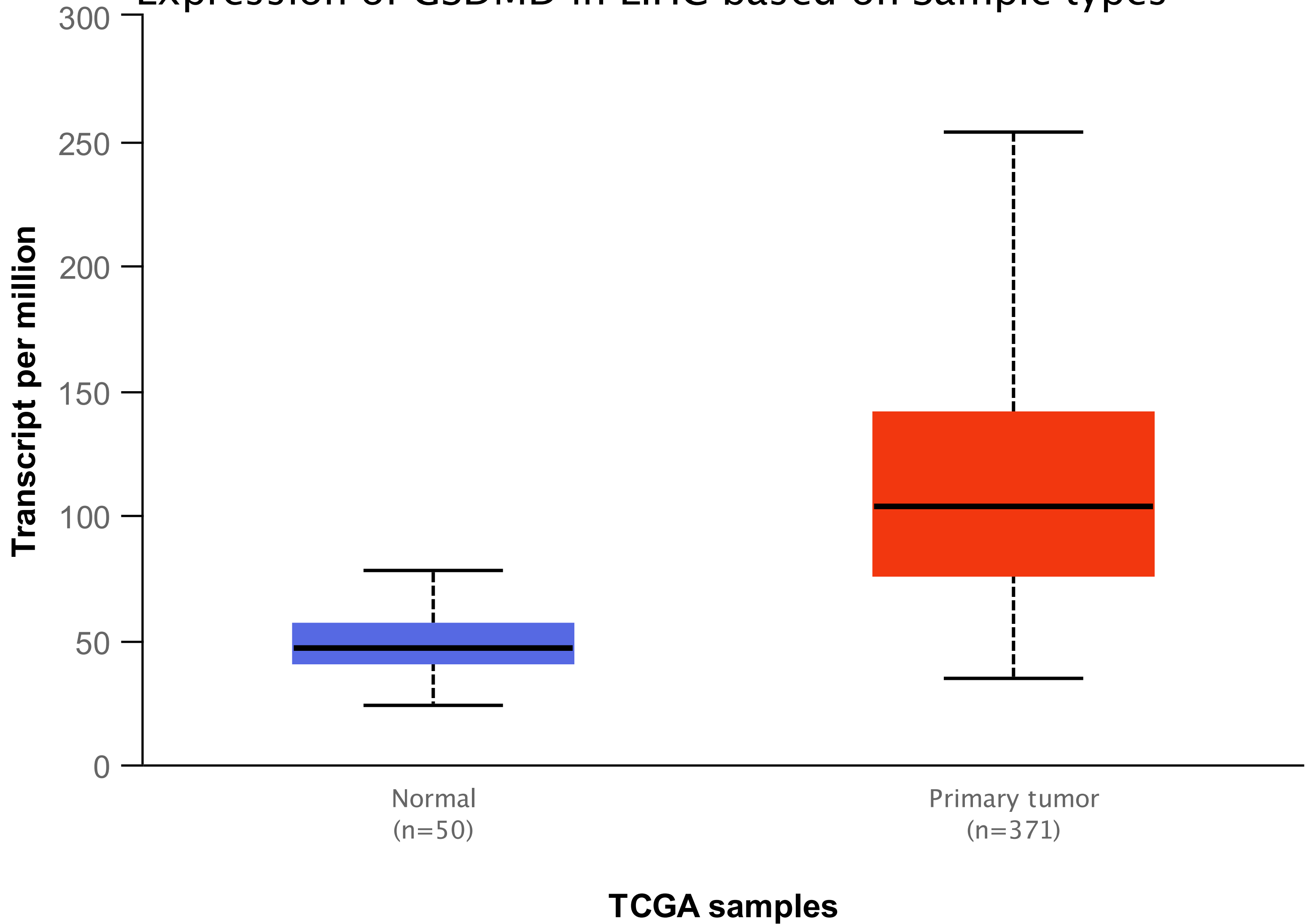

Supplement: Supplementary file 4 [file DataSheet_2.zip › Source data of bioinformatics/GSDMD/expression-of-gsdmd-in-l.pdf]

# Expression of NEK7 in LIHC based on Sample types

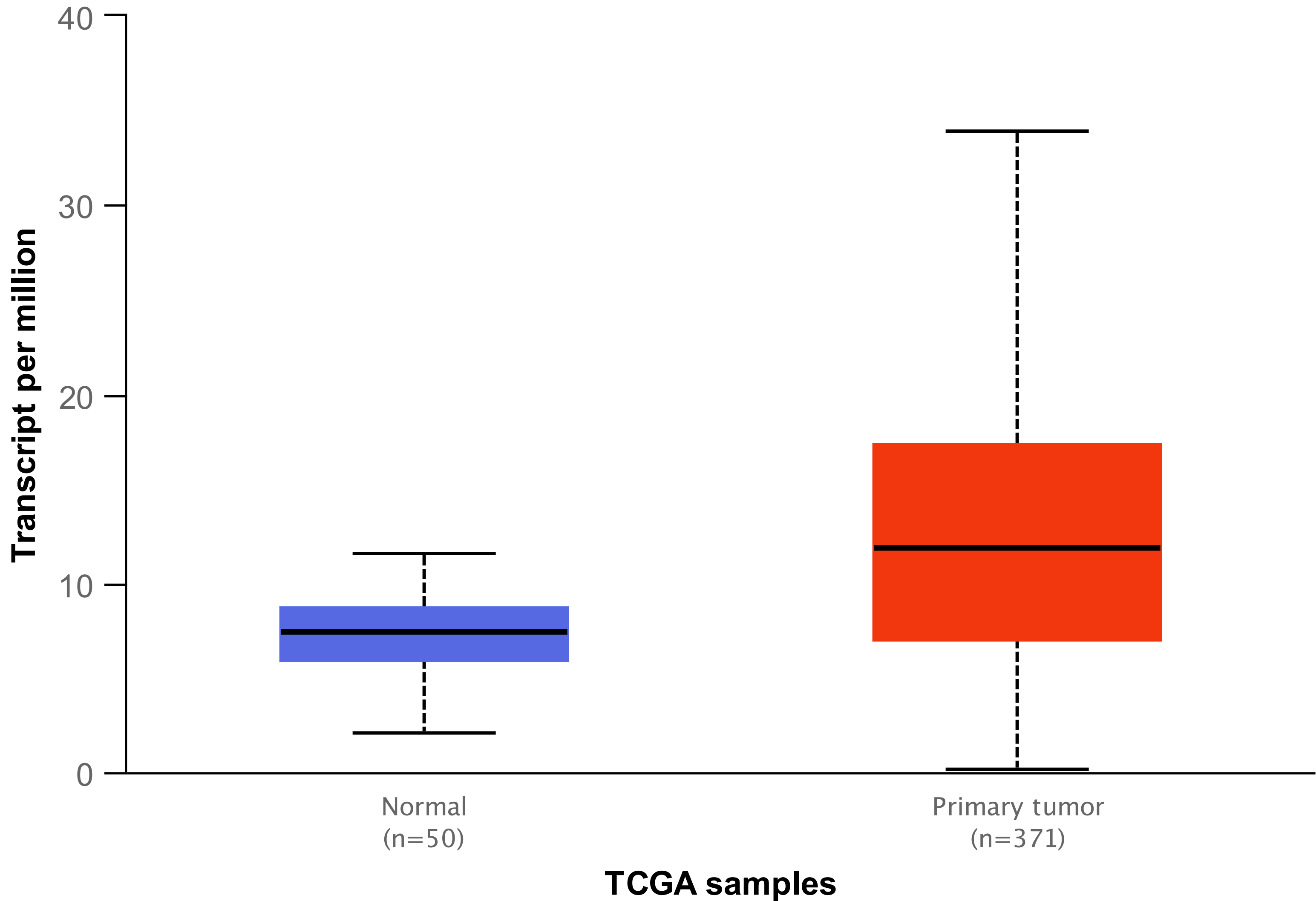

Supplement: Supplementary file 4 [file DataSheet_2.zip › Source data of bioinformatics/NEK7/expression-of-nek7-in-li.pdf]

# Expression of NEK7 in LIHC based on tumor grade

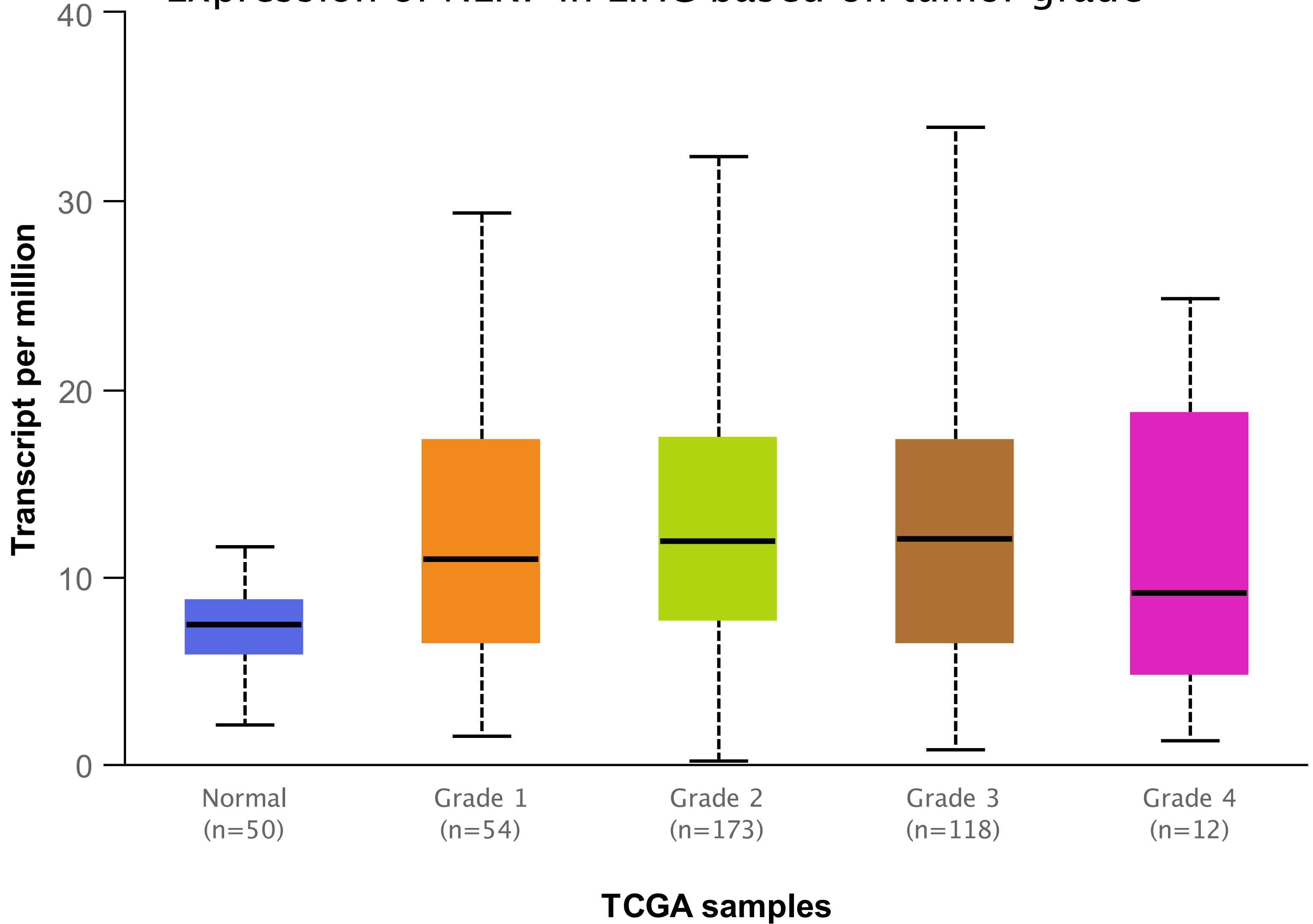

Supplement: Supplementary file 4 [file DataSheet_2.zip › Source data of bioinformatics/NEK7/expression-of-nek7-in-li-3.pdf]

# Expression of NEK7 in LIHC based on individual cancer stages

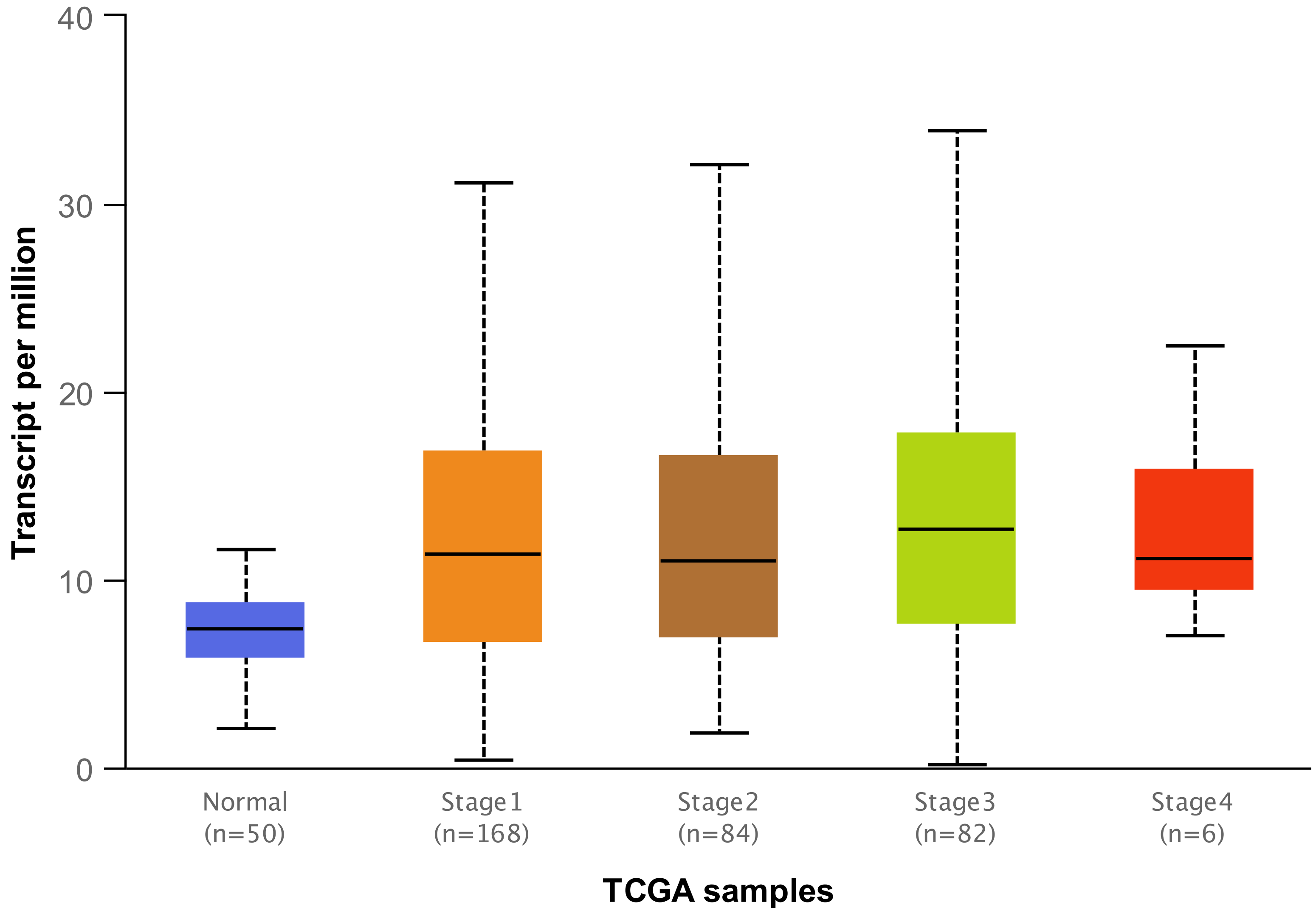

Supplement: Supplementary file 4 [file DataSheet_2.zip › Source data of bioinformatics/NEK7/expression-of-nek7-in-li-2.pdf]

Expression of NEK7 in LIHC based on TP53 mutation status

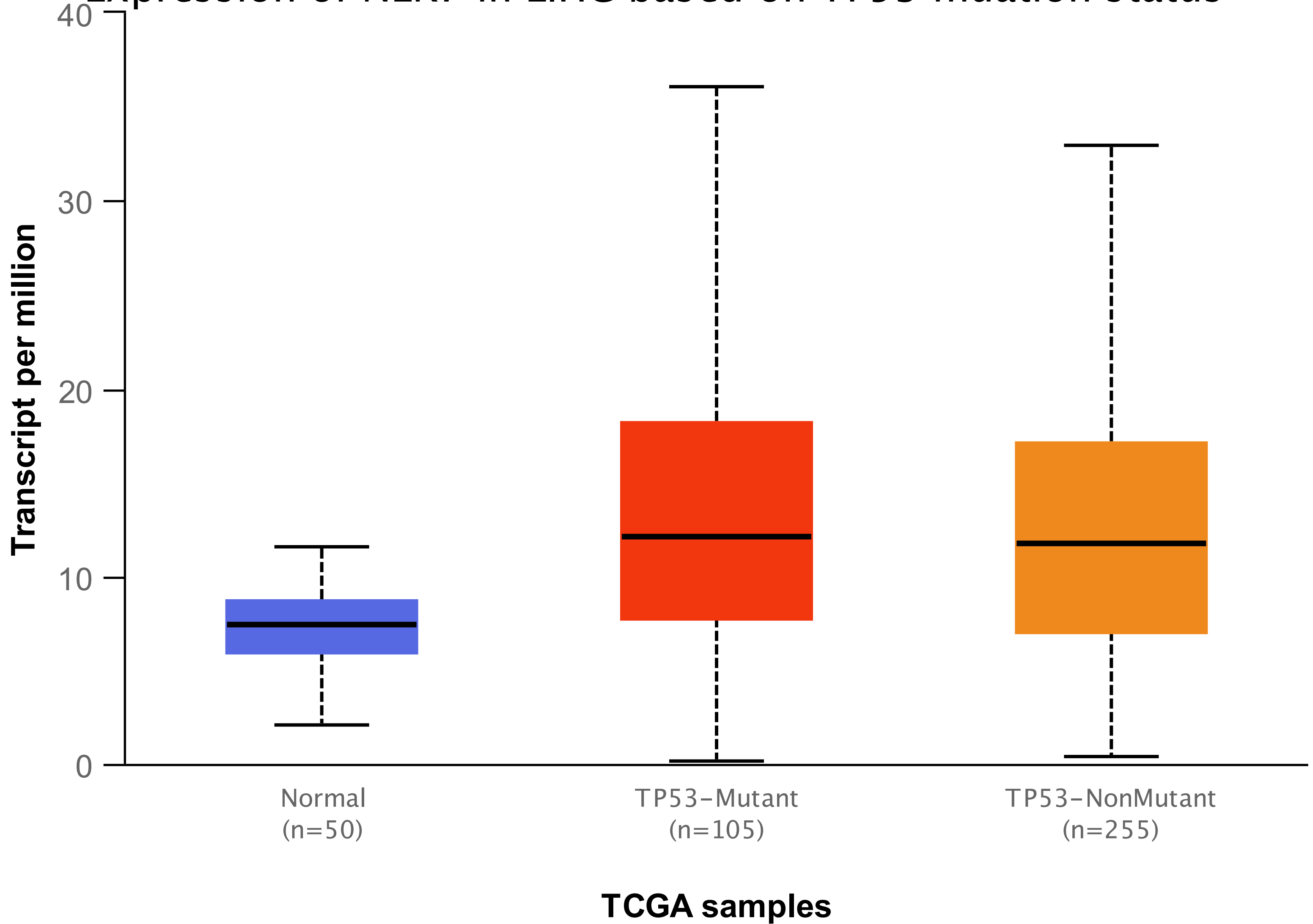

Supplement: Supplementary file 4 [file DataSheet_2.zip › Source data of bioinformatics/NEK7/expression-of-nek7-in-li-5.pdf]

# Expression of NEK7 in LIHC based on nodal metastasis status

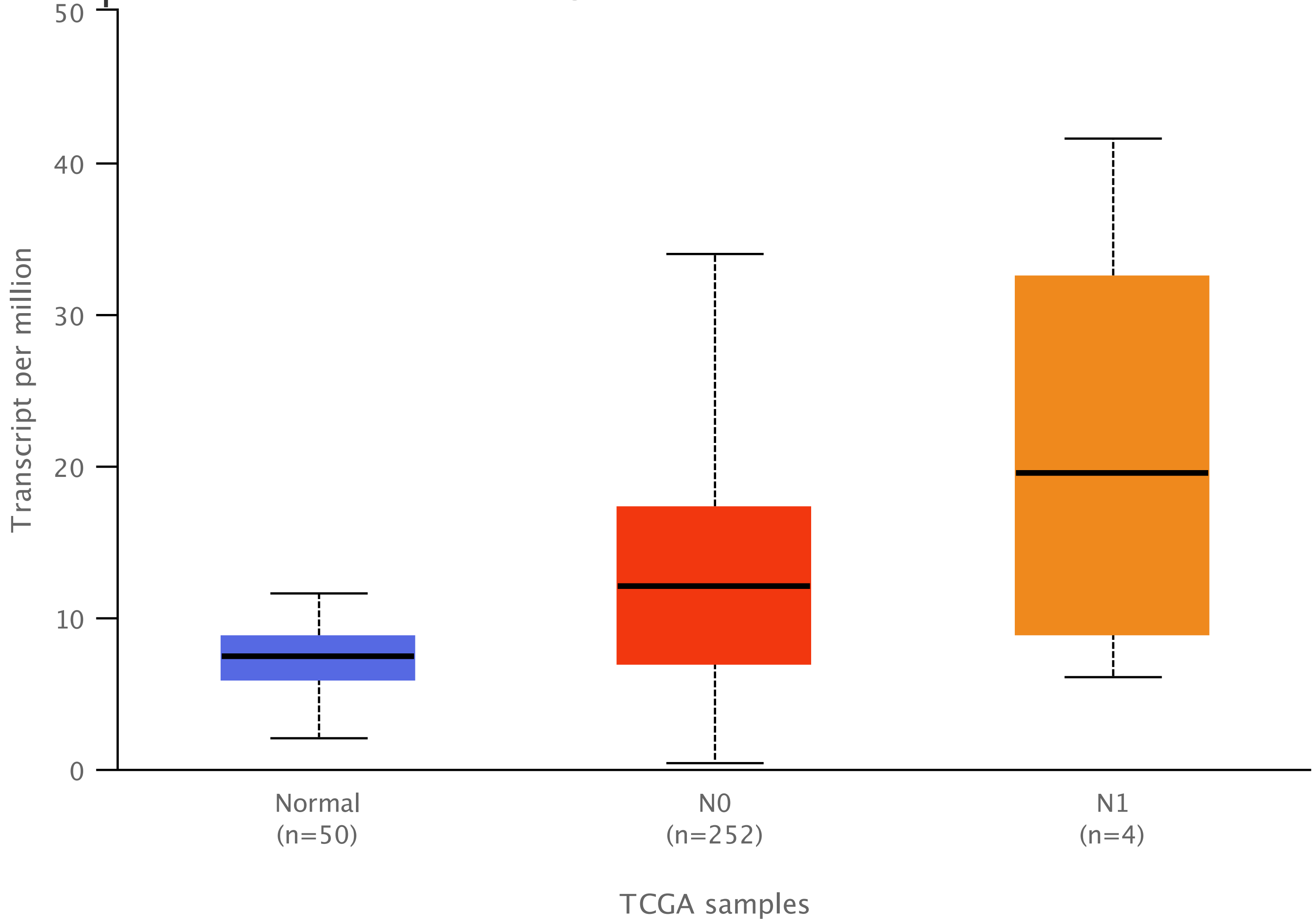

Supplement: Supplementary file 4 [file DataSheet_2.zip › Source data of bioinformatics/NEK7/expression-of-nek7-in-li-4.pdf]

# Expression of NEK7 in LIHC based on Histological subtypes

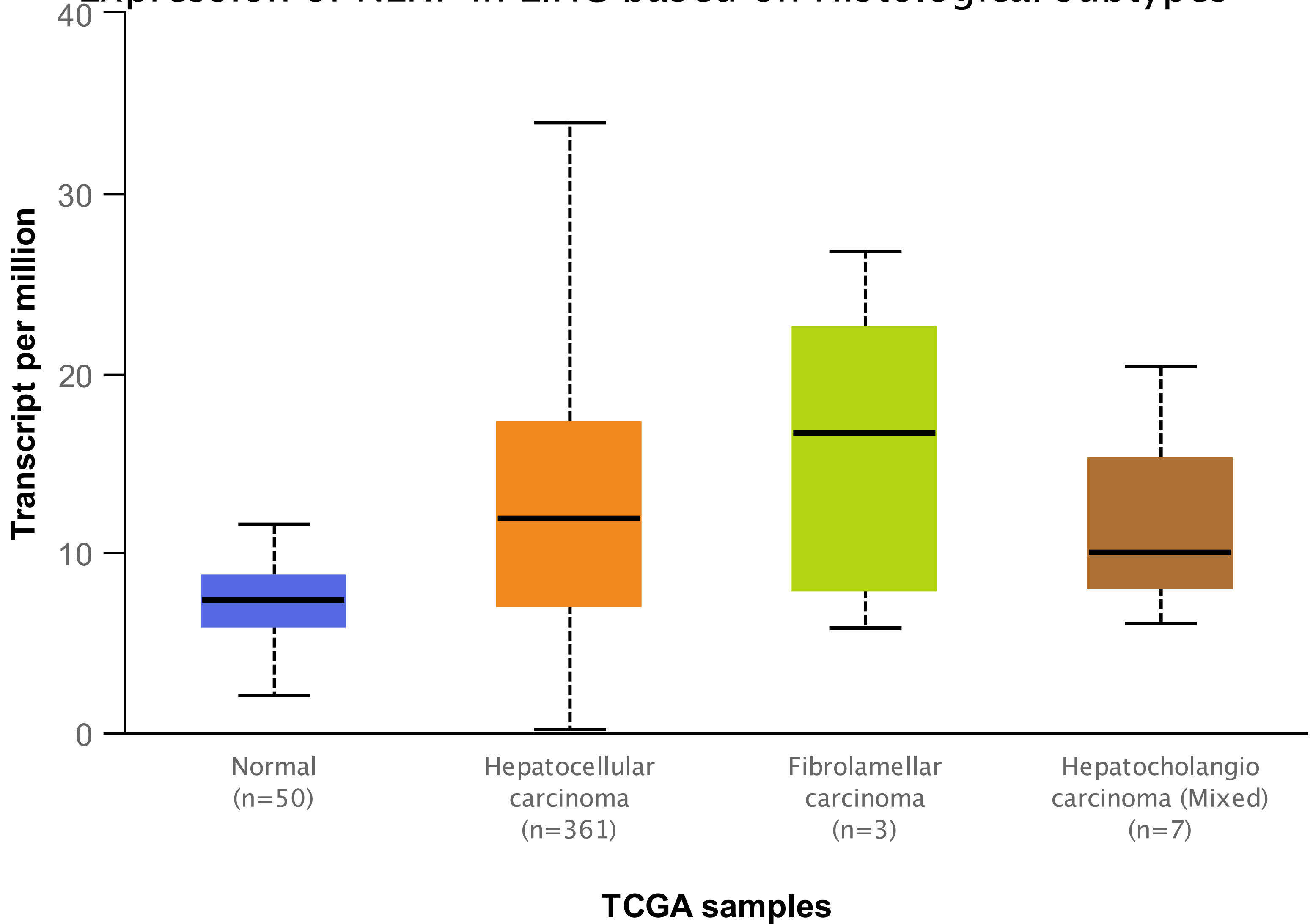

Supplement: Supplementary file 4 [file DataSheet_2.zip › Source data of bioinformatics/NEK7/expression-of-nek7-in-li-6.pdf]
